# ^18^F-C2Am: a targeted imaging agent for detecting tumor cell death in vivo using positron emission tomography

**DOI:** 10.1186/s13550-020-00738-7

**Published:** 2020-12-09

**Authors:** Flaviu Bulat, Friederike Hesse, De-En Hu, Susana Ros, Connor Willminton-Holmes, Bangwen Xie, Bala Attili, Dmitry Soloviev, Franklin Aigbirhio, Finian. J. Leeper, Kevin M. Brindle, André A. Neves

**Affiliations:** 1 Cancer Research UK Cambridge Institute, Li Ka Shing Centre, University of Cambridge, Robinson Way, Cambridge, CB2 0RE UK; 2 Department of Chemistry, University of Cambridge, Cambridge, CB2 1EW UK; 3 Wolfson Brain Imaging Centre, University of Cambridge, Cambridge, CB2 0QQ UK

**Keywords:** Cell death, PET, C2Am, Synaptotagmin-I, Tumor, TRAILR2

## Abstract

**Introduction:**

Trialing novel cancer therapies in the clinic would benefit from imaging agents that can detect early evidence of treatment response. The timing, extent and distribution of cell death in tumors following treatment can give an indication of outcome. We describe here an ^18^F-labeled derivative of a phosphatidylserine-binding protein, the C2A domain of Synaptotagmin-I (C2Am), for imaging tumor cell death in vivo using PET.

**Methods:**

A one-pot, two-step automated synthesis of N-(5-[^18^F]fluoropentyl)maleimide (60 min synthesis time, > 98% radiochemical purity) has been developed, which was used to label the single cysteine residue in C2Am within 30 min at room temperature. Binding of ^18^F-C2Am to apoptotic and necrotic tumor cells was assessed in vitro, and also in vivo, by dynamic PET and biodistribution measurements in mice bearing human tumor xenografts treated with a TRAILR2 agonist or with conventional chemotherapy. C2Am detection of tumor cell death was validated by correlation of probe binding with histological markers of cell death in tumor sections obtained immediately after imaging.

**Results:**

^18^F-C2Am showed a favorable biodistribution profile, with predominantly renal clearance and minimal retention in spleen, liver, small intestine, bone and kidney, at 2 h following probe administration. ^18^F-C2Am generated tumor-to-muscle (T/m) ratios of 6.1 ± 2.1 and 10.7 ± 2.4 within 2 h of probe administration in colorectal and breast tumor models, respectively, following treatment with the TRAILR2 agonist. The levels of cell death (CC3 positivity) following treatment were 12.9–58.8% and 11.3–79.7% in the breast and colorectal xenografts, respectively. Overall, a 20% increase in CC3 positivity generated a one unit increase in the post/pre-treatment tumor contrast. Significant correlations were found between tracer uptake post-treatment, at 2 h post-probe administration, and histological markers of cell death (CC3: Pearson *R* = 0.733, *P* = 0.0005; TUNEL: Pearson *R* = 0.532, *P* = 0.023).

**Conclusion:**

The rapid clearance of ^18^F-C2Am from the blood pool and low kidney retention allowed the spatial distribution of cell death in a tumor to be imaged during the course of therapy, providing a rapid assessment of tumor treatment response. ^18^F-C2Am has the potential to be used in the clinic to assess early treatment response in tumors.

## Introduction

The aim of cancer treatment, whether this is chemo-, radio-, targeted- or immuno-therapy [1], is to induce tumor cell death, where the two dominant forms of cell death are apoptosis and necrosis [2]. Cell death is an important and generic target for imaging early treatment response [3]. However, despite a long-standing unmet need there are still no reliable techniques for routine imaging of cell death in the clinic [4].

Phosphatidylserine (PS) is externalized on the cell surface during apoptosis and is also exposed via the permeabilization of the plasma membrane that occurs during necrosis. The C2A domain of Synaptotagmin-I binds PS in a calcium-dependent manner with nanomolar affinity [5, 6]. We have developed a PS-targeted imaging agent based on C2A, which was first used in vivo as a glutathione S-transferase–tagged dimeric construct (GST-C2A, 84 kDa) for imaging tumor cell death using MRI, where the protein was labeled with superparamagnetic iron oxide nanoparticles [7] and subsequently with Gd^3+^ chelates [8]. This GST-tagged construct has also been labeled with a ^99m^Tc-chelate for SPECT [9] and with fluorine-18 for PET [10].

Subsequently, we have used the much smaller (16-kDa) isolated C2A domain [6], which enables better target access and tissue clearance. Introduction of a single cysteine residue, distant from the PS-binding site, using site-directed mutagenesis (S78C; C2Am), allowed the production of chemically defined derivatives in which this single cysteine was labeled with imaging tags. A near infrared-labeled derivative showed a fourfold lower binding to viable cells in vitro than a similarly labeled Annexin-V and therefore improved specificity for detecting cell death [6]. Annexin-V is another PS-binding protein, which was tested in the clinic nearly two decades ago in the form of ^99m^Tc-HYNIC-Annexin-V, but showed suboptimal pharmacokinetics and extensive non-specific binding [11, 12]. A subsequent derivative, ^99m^Tc-rh-Annexin-V-128, showed a better biodistribution profile and targeting of cell death in vivo [13].

We recently evaluated derivatives of C2Am that had been labeled for photoacoustic imaging [14] and SPECT [15]. The latter were based on ^99m^Tc and ^111^In chelates and could detect tumor cell death in vivo within 2 h of administration, with a tumor-to-muscle contrast of ~ 3. However, they also showed significant renal retention (> 150% IA/g), which persisted for up to 24 h post-administration.

Here, we describe a ^18^F-labeled derivative of C2Am that was tested in human xenograft models of advanced colorectal (Colo205 [16]) and triple-negative breast (MDA-MB-231 [17]) cancer, treated with either conventional chemotherapy or with MEDI3039, which is a multivalent tumor necrosis factor (TNF)-related apoptosis-inducing ligand receptor-2 (TRAILR2) agonist that can induce tumor cell death at picomolar concentrations [18]. We analyzed the biodistribution and dosimetry profile of the probe and its sensitivity for detecting tumor cell death by correlating probe distribution in tumors with histological markers of tumor cell death in tumor sections. Non-specific retention of the probe was evaluated by comparing its distribution with that of a site-directed mutant, ^18^F-iC2Am, which is inactive in PS binding [15].

## Materials and methods

### General

Details of the materials used including the precursor N-(5-[^18^F]fluoropentyl)maleimide and the imaging probe ^18^F-C2Am can be found in Additional file 1.

### Cell culture

Chemicals were obtained from Sigma-Aldrich unless stated otherwise. MDA-MB-231 (ATCC, HTB-26™) and Colo205 (ATCC, CCL-222™) were purchased and stably transfected with firefly luciferase for bioluminescence imaging (BLI) using a method described previously [19], and used within ten passages from the original stocks. Both cell lines tested negative for mycoplasma and were authenticated using short-tandem repeat genetic profiling [20] yielding a 100% match to the cell lines in the ATCC database. MDA-MB-231 and Colo205 cells were cultured in DMEM or RPMI-1640 medium (Life Technologies), respectively, supplemented with 2 mmol/L l-glutamine and 10% fetal bovine serum (Life Technologies), in a humidified incubator at 37 °C and 5% CO_2_.

### Flow cytometry

Colo205 and MDA-MB-231 cells (2 million) were treated with 2.5 pM and 10 pM MEDI3039, respectively, for 24 h, centrifuged (700*g*, 5 min, 4 °C) and resuspended in 100 μL of pre-cooled HBS buffer (HEPES-buffered saline: 10 mmol/L HEPES, 150 mmol/L NaCl, 2 mmol/L CaCl_2_, 1% fetal bovine serum (FBS), pH 7.4). Following incubation with SYTOX-Red (Invitrogen, 50 nM) and with C2Am-AF750 or AnnexinV-AF647 (conjugated to Alexa Fluor 750 or 647, respectively), at concentrations of 0.5 μM and 20 nM, respectively, the cells were washed twice, resuspended and kept on ice before analysis in a Symphony cytometer (Beckman), with 50,000 cells counted per event. Data were analyzed using FlowJo software (vs. 10) using methods described previously [6].

### Cell labeling with ^18^F-C2Am

Vehicle- and MEDI3039-treated (10 pM, 24 h) cells (*n* = 3) were harvested, counted and viability assessed using a Vi-CELL analyzer (Beckman). The cells were then resuspended in HBS buffer and incubated with [^18^F]FPenM-C2Am (1–10 μM, 7–11 MBq, Am = ~ 10.5 GBq/µmol at the start of labeling) at 37 °C for 20 min. Cell pellets (5 million cells, centrifuged at 700*g* for 5 min at 4 °C) were washed three times with HBS buffer (1 mL) and radioactivity counted for 1 min using a gamma counter (AMG Hidex) set to monitor the fluorine-18 gamma emission (511 keV).

### Animal studies

Animal experiments were performed in compliance with a project license issued under the Animals (Scientific Procedures) Act of 1986 and were designed with reference to the UK Co-ordinating Committee on Cancer Research guidelines for the welfare of animals in experimental neoplasia [21]. Protocols were approved by the Cancer Research UK Cambridge Institute Animal Welfare and Ethical Review Body. Colo205 or MDA-MB-231 cells (5 or 10 million, respectively) were resuspended in 0.1 mL PBS or a 1:1 mixture of Matrigel (Corning) and complete DMEM, respectively, and implanted subcutaneously in the upper back of 10–12 week-old female BALB/c Nu/Nu mice (Charles River). Tumors were imaged when they reached ~ 1 cm^3^. For PET/CT imaging, mice were anesthetized using 1–2.5% isoflurane (Isoflo, Abbotts Laboratories Ltd.) in a 1:1 mixture of air and oxygen (at 1 L/min). MEDI3039, a TRAILR2 agonist, was administered at 0.1–0.4 mg/kg (i.v.) [22]. 5-fluorouracil (5FU) and doxorubicin (DOX) were used at 50–100 mg/kg (i.p.) and 100 mg/kg (i.p.), in the Colo205 and MDA-MB-231 models, respectively.

### Production of ^18^F-C2Am

**Fig. 1:**
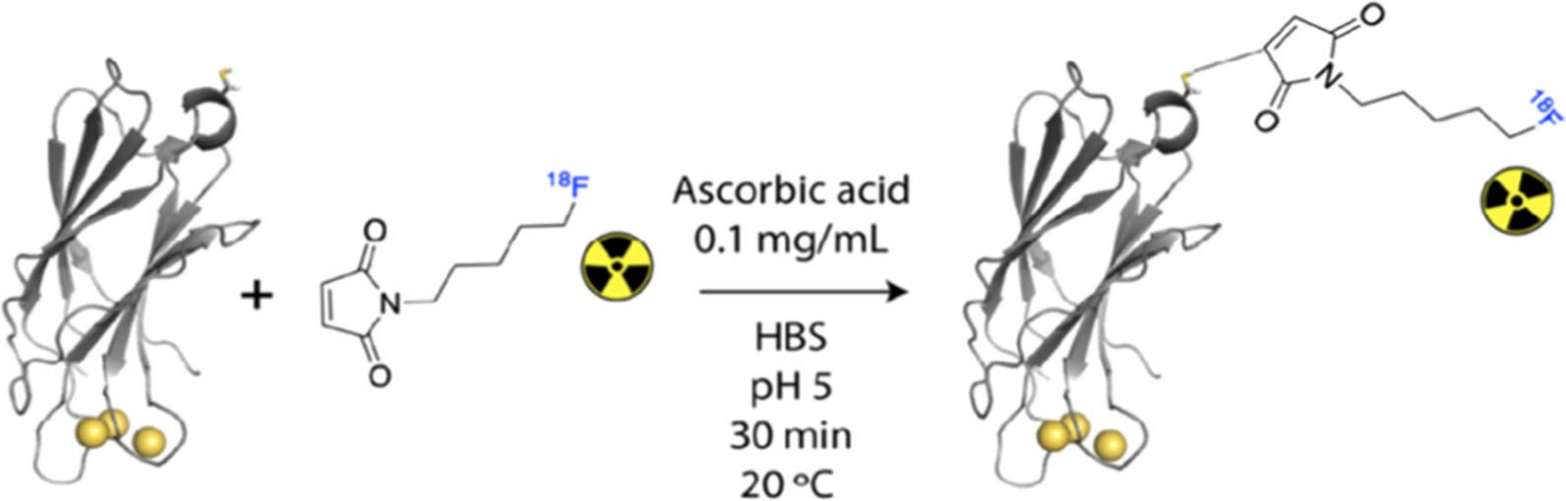
Radiochemical labeling of C2Am, using N-(5-[^18^F]fluoropentyl) maleimide, to yield ^18^F-C2Am. The rapid Michael addition reaction takes place under mild conditions (20 °C, pH5, HBS buffer) in the presence of ascorbic acid. The secondary structure of C2Am is shown, highlighting the single cysteine residue (top, yellow) and the active site (lower) containing three calcium ions (yellow spheres)

A one-pot, two-step automated synthesis (Additional file 1: Fig. S1) of the prosthetic group N-(5-[^18^F]fluoropentyl) maleimide was developed using an automated module (GE TracerLab FxFN, 60 min synthesis time, > 98% radiochemical purity and 12 ± 3% decay corrected yield, *n* = 3; Additional file 1: Fig. S2). This was used to fully label the single cysteine residue in C2Am (Fig. 1) within 30 min at 20 °C (Am = 212 ± 30 GBq/µmol, > 95% radiochemical purity, *n* = 3, Fig. 1 and Fig. S3) to give ^18^F-C2Am. Details on the radiosynthesis procedure, quality control and radiometabolite analysis methods can be found in Additional file 1.

### Dynamic ^18^F-C2Am PET/CT imaging of treatment response

Colo205 (*n* = 9) and MDA-MB231 (*n* = 9) tumor–bearing mice underwent bioluminescence imaging (BLI) and PET-CT, following injection of ^18^F-C2Am, which were performed in the same 2.5 h imaging session before and 24 h following treatment with MEDI3039 or chemotherapy (5FU or DOX, respectively). Following BLI, helical CT data were acquired for anatomical reference and attenuation correction (isotropic resolution of 0.2 mm). PET images, with a nominal isotropic resolution of 0.6 mm, were reconstructed using a 3D ordered subset expectation maximization (OSEM) method in one to three coincidence modes, eight iterations and six subsets. A 120-min dynamic PET acquisition was initiated 30 s prior to intravenous injection of 3.2 ± 1.3 MBq ^18^F-C2Am (150 μg/kg; 10 mL/kg; S_A_ = 1 MBq/μg), using a nanoScan PET/CT (Mediso) scanner. Scans were reconstructed into 23 time frames (4 × 15 s, from 0–1 min; 4 × 1 min, 1–5 min; 11 × 5 min, 5–60 min; 4 × 15 min, 60–120 min). Images were normalized and corrected for decay and attenuation and analyzed using VivoQuant software (vs. 3, InviCRO). Three-dimensional tissue regions of interest (ROI) were drawn manually, and, if possible, Otsu thresholding was applied to better delineate the ROIs.

Standardized uptake values (SUV) were calculated as mean (SUV), median (SUV_50_), the 90th percentile (SUV_90_), peak (SUV_peak_), peak_max (SUV_peakM_) and maximum (SUV_max_). SUV (g/mL) was defined as SUV = C_img_/(IA/BW), where C_img_ is the activity concentration (MBq/mL) in the ROI, IA is the injected activity (MBq), and BW is the body weight of the animal (in grams). The pixel with the maximum signal intensity was used to calculate SUV_max_. The fraction of injected activity per gram of tissue (IA/g, %), the tumor-to-muscle (T/m) and tumor-to-blood (T/b) ratios were also calculated, the latter two using the lower flank skeletal muscle and carotid artery, respectively. Additional details are available in the Additional file 1.

## Results

### Flow cytometry of dying cells using C2Am-AF750

**Fig. 2:**
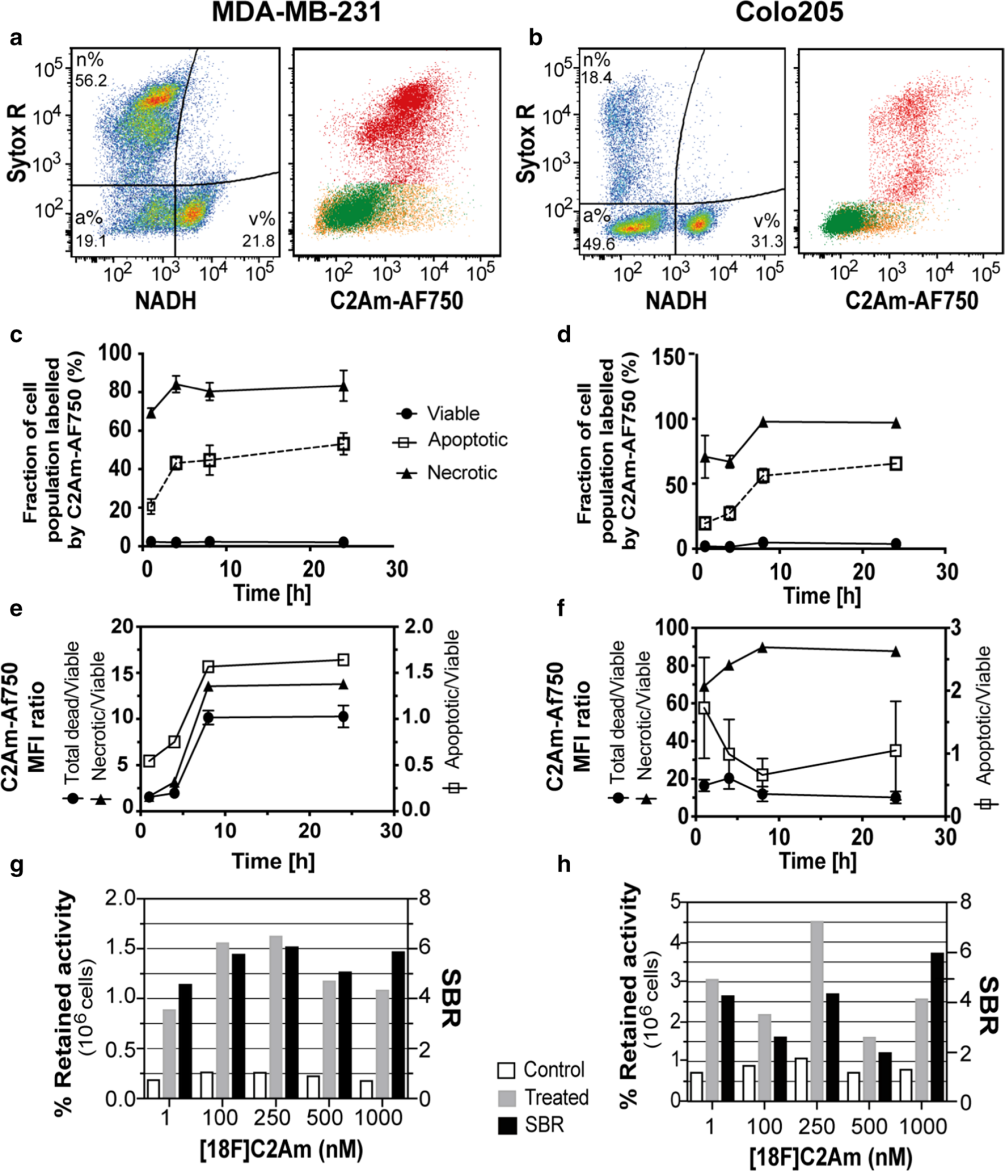
C2Am binds to dead cells in vitro. Flow cytometric analysis of MEDI3039-treated MDA-MB-231 (**a**) and Colo205 (**b**) cells shows three distinct populations (first and third plots, from left), which were gated based on their levels of UV_A_ autofluorescence (NADH content) and plasma membrane integrity (Sytox R) as: viable (v%), apoptotic (a%) and necrotic (n%) cells. C2Am-AF750, a near infrared fluorophore-labeled derivative of C2Am labels (second and fourth plots) preferentially bound apoptotic (orange) and necrotic (red) cells, in comparison with viable (green) cells (**c**, **d**). Plots of C2Am-AF750 mean fluorescence intensity (MFI) ratios for necrotic/viable, apoptotic/viable and dead/viable MDA-MB-231 (**e**) and Colo205 (**f**) cells following treatment with MEDI3039 (mean ± SD, *n* = 3), error bars lie within the symbols when not shown. MEDI3039-treated MDA-MB-231 (**g**) and Colo205 (**h**) cells were incubated with ^18^F-C2Am at different concentrations (1–1000 nM) and the fraction of activity retained in the cell pellets (%) after three washes was measured. The signal-to-background ratio (SBR, black bars) of treated (grey bars) versus vehicle-treated (open bars, control) cells is also shown

Following treatment with MEDI3039, MDA-MB-231 and Colo205 cells were predominantly necrotic (56.2%) or apoptotic (49.6%), respectively (Fig. 2a, b), based on cell membrane integrity, determined by Sytox Green staining, and the levels of NADH, which have been demonstrated previously as independent markers of these two modes of cell death [6]. Both cell lines upregulated expression of the TRAILR2 receptor upon treatment with the agonist (Additional file 1: Fig. S4). Necrotic and apoptotic cells showed greater mean fluorescence intensity (MFI) with C2Am-AF750 than viable cells, for both cell lines (Fig. 2c, d). Overall, the C2Am-AF750 MFI ratio for dead/viable cells (Fig. 2e, f, closed circles) at 24 h after treatment was similar for both cell lines (~ 10). Cell staining using Annexin-V (Fig. S5) indicated similar levels of apoptotic and necrotic cells, as those determined with C2Am, albeit the labeling of viable MDA-MB-231 and Colo205 cells using Annexin-V was greater than that observed for C2Am, as reported previously [6]. Incubation of ^18^F-C2Am with the cells showed ~ 4-fold greater retention of activity by MEDI3039-treated cells, in comparison with vehicle-treated control cells (see signal-to-background ratio (SBR) in Fig. 2g, h). The greater retention of ^18^F-C2Am by MEDI3039-treated Colo205 cells (Fig. 2h) implies that there was greater exposure of PS in the predominantly apoptotic Colo205 cells as opposed to the predominantly necrotic MDA-MB-231 cells (Fig. 2a, b). Treatment of the Colo205 cells produced a large number of small apoptotic bodies (∼8 μm vs 14 μm diameter for intact cells) and failure to fully capture these by centrifugation may have contributed to the variation observed in ^18^F-C2Am retention in the cell pellets (Fig. 2h).

### Biodistribution of ^18^F-C2Am

**Fig. 3:**
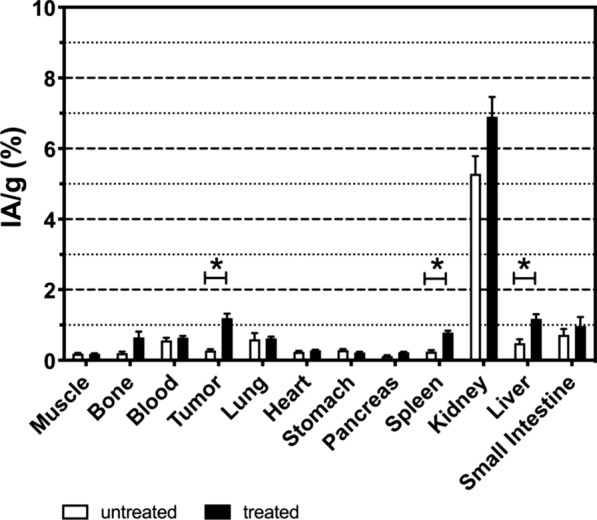
Biodistribution profile of ^18^F-C2Am, 2 h post-administration. Mice bearing Colo205 tumors were treated with MEDI3039 (closed bars; 0.4 mg/kg, i.v., 24 h) or vehicle (open bars). ^18^F-C2Am was injected (1 MBq, i.v.) and tissues collected post-mortem 2 h following administration. Fraction of injected activity per gram of tissue (IA/g, %) (mean ± SEM, *n* = 5 per treatment group). Two-tailed, *t* test, unequal variance. *P < 0.05

Biodistribution was assessed in MEDI3039- and vehicle-treated control mice bearing Colo205 tumors (Fig. 3). Two hours following ^18^F-C2Am administration most tissues showed low levels of activity (< 2% IA/g). Tumor activity post-treatment was at least ∼ 2-fold greater than in every other organ with the exception of kidney (the main clearance route). Renal retention (∼ 6% IA/g) was low and identical in both cohorts. The uptake of ^18^F-C2Am was significantly higher (*P* < 0.05) in tumor, spleen and liver tissue following treatment, albeit the increase was smaller (~ 2.5 ×) in spleen and liver, compared with tumor (~ 5 ×) (Fig. 3). There was increased cell death in the spleen (*P* < 0.001) and liver following MEDI3039 treatment, although the increase in the liver was not significant (Additional file 1: Fig. S6), which is likely due to low-level expression of TRAILR2 in these tissues [23]. The blood half-life of ^18^F-C2Am, calculated from the analysis of dynamic data obtained from ROIs placed in the carotid artery, was 12.4 ± 2.2 min. ^18^F-C2Am was stable in plasma for up to 8 h at 37 °C (Additional file 1: Fig. S7) and was found to be intact in mouse blood in vivo at 15 min post-administration (Additional file 1: Fig. S8A), although by 30 min a lower molecular weight metabolite (< 3 kDa) had appeared and by 60 min approximately half of the radioactivity present was in this metabolite (Additional file 1: Fig. S8C-D). The same metabolite also appeared in the urine at 15 min and its concentration increased further at 30 and 60 min (Additional file 1: Fig. S8B, C-D), suggesting that there is some loss of the prosthetic group in vivo.

### Dynamic PET imaging of tumor cell death using ^18^F-C2Am

**Fig. 4:**
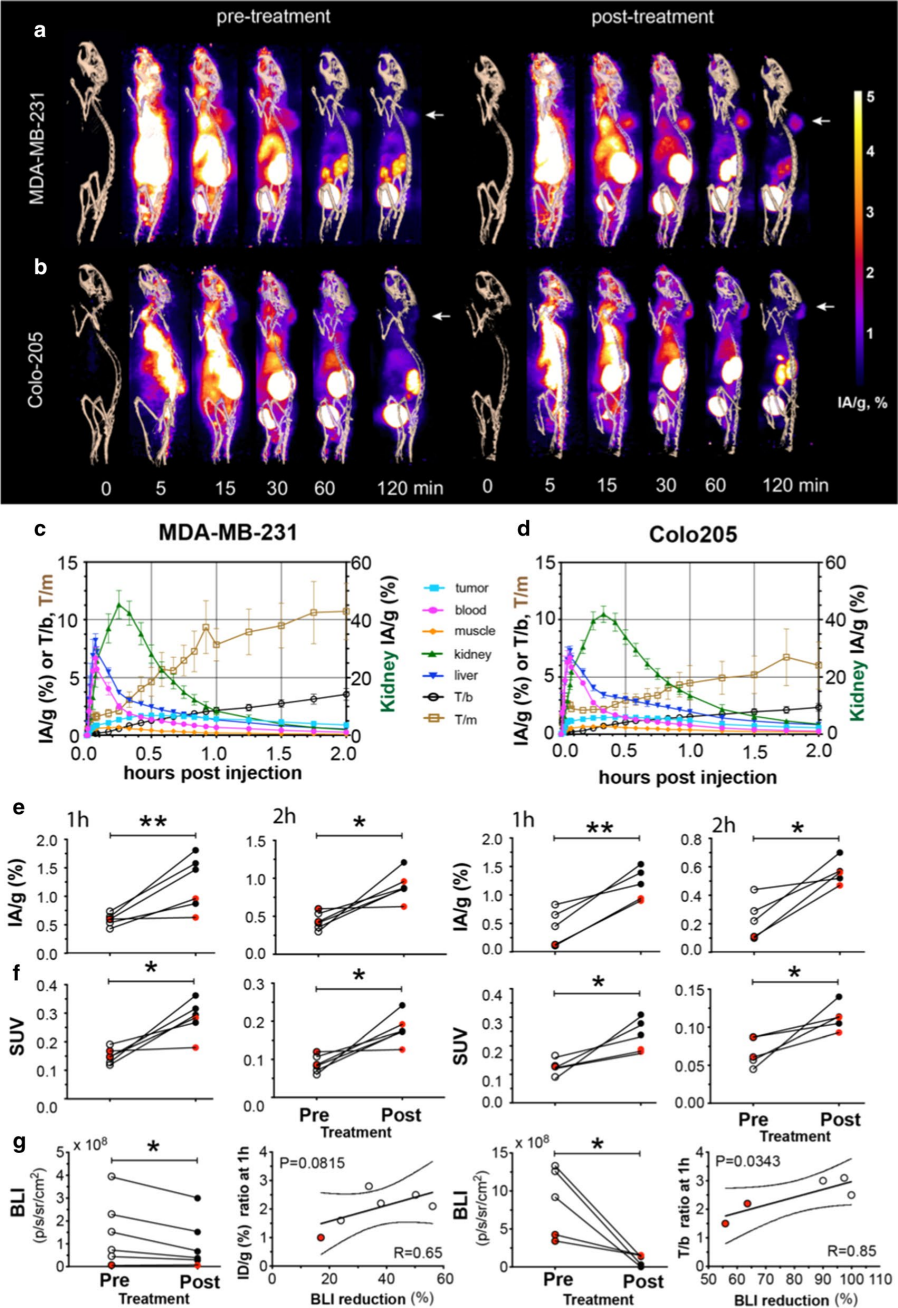
PET images of ^18^F-C2Am in tumor-bearing mice, pre- and post-treatment. Maximum intensity projections of the PET signal in sagittal sections of representative mice, bearing MDA-MB-231 (**a**) and Colo205 (**b**) tumors. Projections are shown from immediately before (0) and up to 120 min after injection of ^18^F-C2Am. Signal is shown as injected activity per gram of tissue (IA/g, %) and overlaid on a CT-derived skeleton mask. White arrows indicate tumor location. Time–activity (IA/g, %) curves for the indicated tissues in treated (MEDI3039, 0.4 mg/kg, 24 h) MDA-MB-231 (**c**) and Colo205 (**d**) tumor-bearing mice. Also shown are tumor-to-blood (T/b) and tumor-to-muscle (T/m) ratios for both tumor models. **e**,** f** Pairwise analysis of PET signal pre- and 1 h (first and third columns) or 2 h (second and fourth columns) post-treatment, expressed as IA/g (%) (**e**) or SUV (**f**). Pairwise analysis (**g**) of tumor bioluminescence pre- and post-treatment (first and third plots) and correlation analysis (Pearson) of tumor PET signal with the reduction in BLI signal (second and fourth plots), 1 h after ^18^F-C2Am administration. Data (**c**, **d**) is mean ± SEM, *n* = 5−6 per treatment group. Black and red symbols (**e**, **g**) correspond to MEDI3039 treatment at 0.4 or 0.1 mg/kg (for 24 h), respectively. **e**, **g** Two-tailed, pairwise *t* test, unequal variance. *P < 0.05, **P < 0.01

Dynamic PET imaging (Fig. 4a, b) confirmed renal excretion as the predominant clearance route for ^18^F-C2Am. Bladder signal could be detected within 5–10 min of administration (Fig. 4a, b) and kidney cortical uptake peaked (at ~ 40% IA/g) within 20–30 min of injection (Fig. 4c, d, green line), but was minimal at 2 h post-administration (~ 3% IA/g; Additional files 2, 3: videos 1 and 2). Tumor signal (Fig. 4a,b) could be detected within 15 min of injection, and there was a small component (< 10%) of hepatobiliary clearance (Fig. 4c, d, blue line). Tumor-to-muscle (T/m) and tumor-to-blood (T/b) ratios were 10.7 ± 2.4 and 3.6. ± 0.5, respectively, for MDA-MB-231 tumors and 6.1 ± 2.1 and 2.4 ± 0.4, respectively, for Colo205 tumors, at 2 h post-administration of ^18^F-C2Am. Analysis of individual tumors before and after treatment (Fig. 4e) showed significant increases in tumor retention (IA/g, %) of ^18^F-C2Am at 1 h (*P* < 0.005) and 2 h (*P* < 0.05) post-administration, for both models. This significant difference (*P* < 0.05) between pre- and post-treatment ^18^F-C2Am tumor uptake was also observed using SUV as the contrast metric (Fig. 4f). The tumor cells had been transduced with a viral vector expressing firefly luciferase prior to implantation, and both tumor models showed a significant decrease in bioluminescence (*P* < 0.05) post-treatment, where this decrease correlated with increased tumor uptake of ^18^F-C2Am in MDA-MB-231 (IA/g, %) and in Colo205 (T/b) tumors (Fig. 4g). The levels of ^18^F-C2Am tumor contrast (SUV and SUV_max_; Fig. S9), obtained 1 h post-administration, were 0.24 ± 0.14 and 0.66 ± 0.18, respectively. There was a dose-dependent response to TRAIL2 agonist therapy in treated tumors for both models (Additional file 1: Fig. S10).

**Fig. 5:**
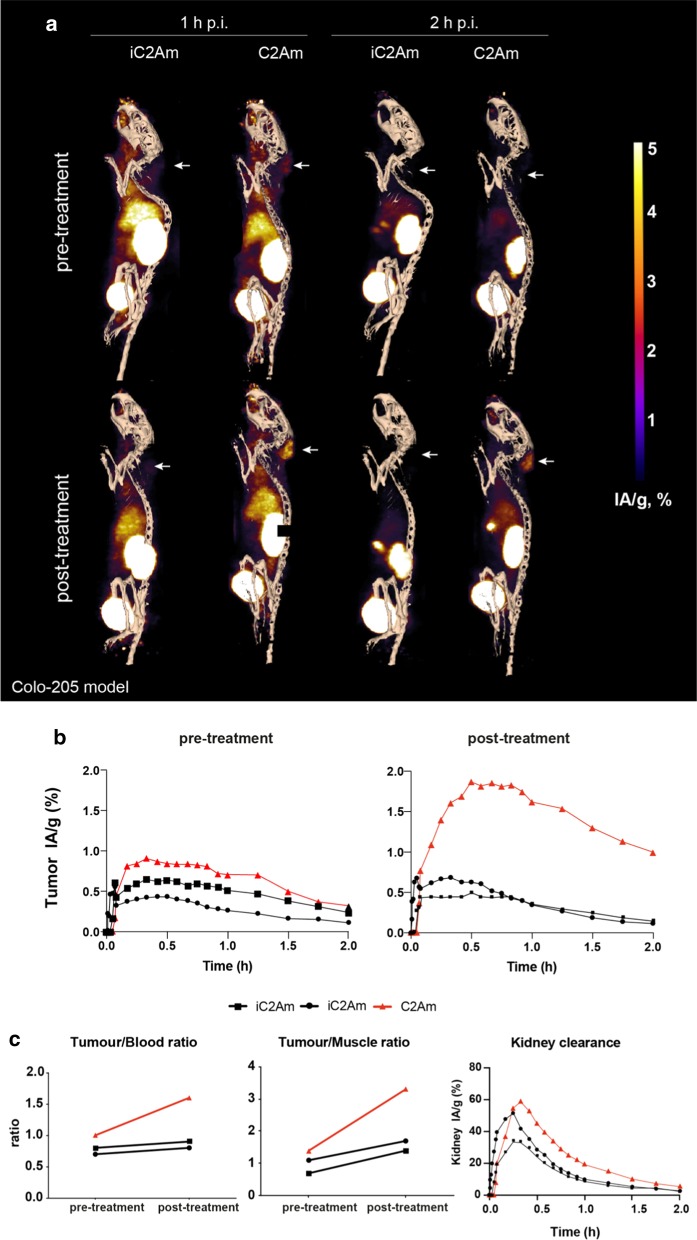
Reduced tumor uptake of (inactive) ^18^F-iC2Am versus (active) ^18^F-C2Am in MEDI3039-treated mice (0.4 mg/kg, 24 h, i.v.) bearing Colo205 tumors. Red lined triangles (^18^F-C2Am); black lined squares (^18^F-iC2Am)

**Fig. 6:**
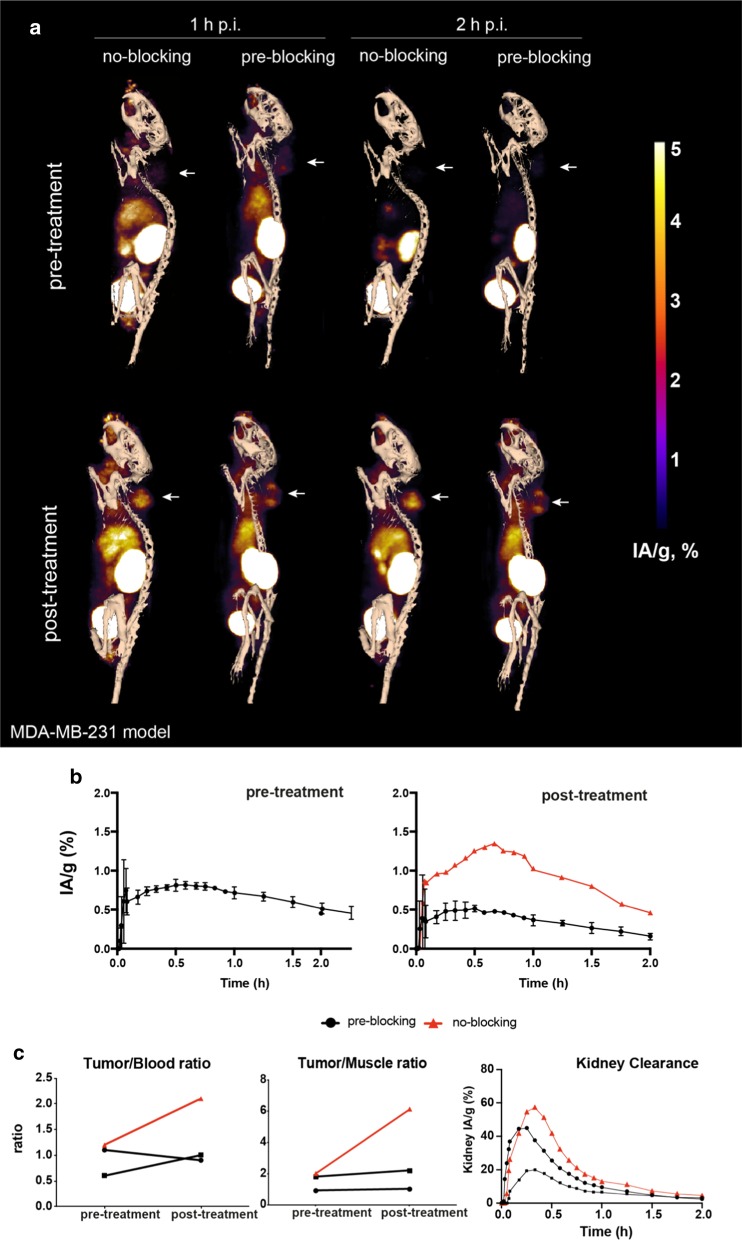
Effect of pre-blocking of PS using (× 10) molar excess of unlabeled C2Am on ^18^F-C2Am tumor contrast in MEDI3039-treated mice (0.4 mg/kg, 24 h, i.v.) bearing MDA-MB-231 tumors. Red lined triangles (^18^F-C2Am; no pre-blocking); black lined squares (^18^F-C2Am; 10x pre-blocking with C2Am)

Only ~ 15% of the contrast obtained with ^18^F-C2Am was observed with ^18^F-iC2Am (Fig. 5). Using unlabeled C2Am as a pre-blocking agent, at 10 × higher molar dose (1.5 mg/kg), generating a C2Am concentration of ~ 30 nM in the tumor, resulted in a ~ 70% decrease of ^18^F-C2Am retention in drug-treated tumors (Fig. 6c).

### Correlation of ^18^F-C2Am with cell death markers

**Fig. 7:**
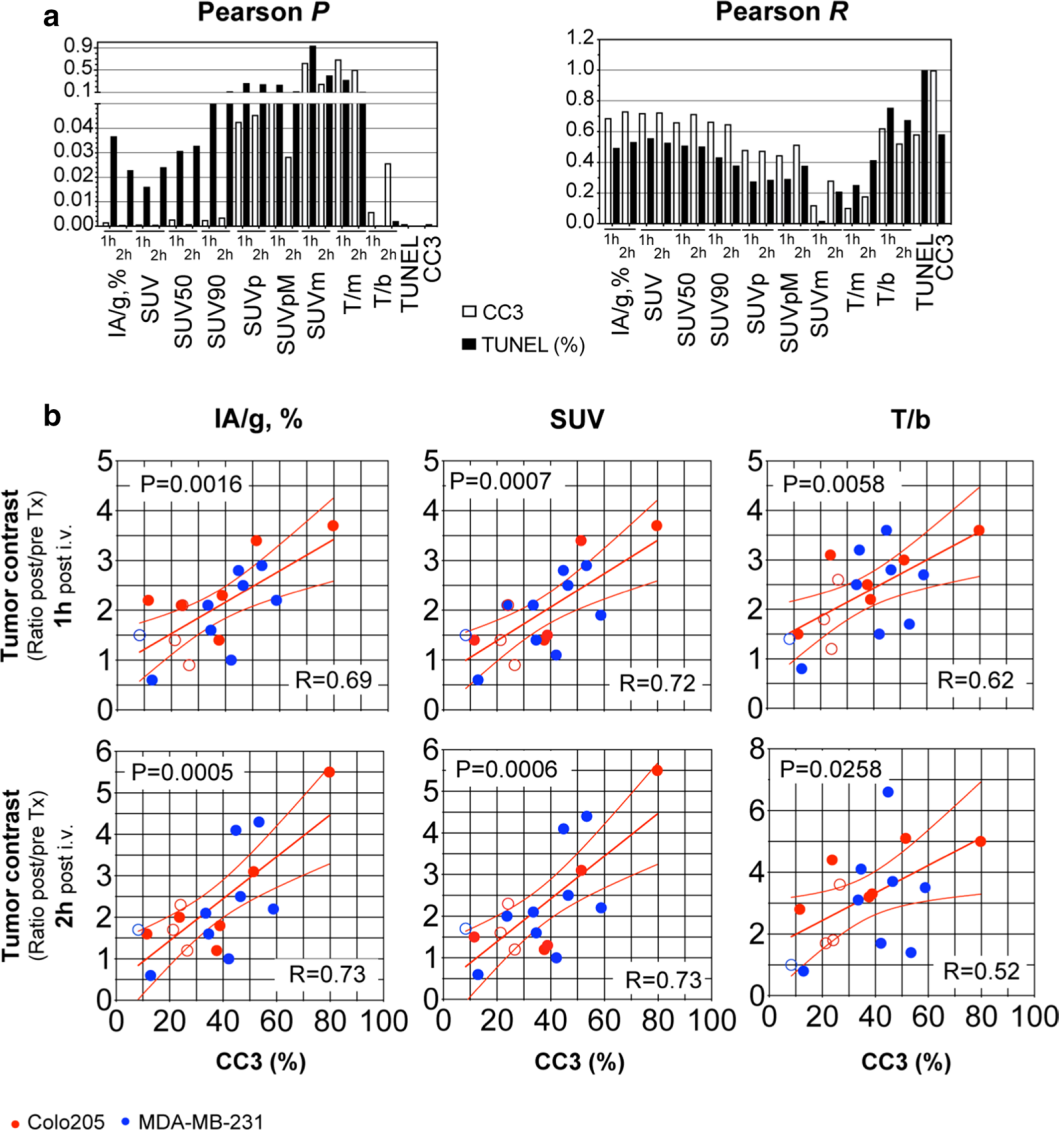
Pearson correlation analysis of tumor PET signal with histological markers of cell death. **a** Pearson *P* (left) and *R* (right) correlation values for tumor signal, expressed as different contrast metrics, 1 h and 2 h post-administration of ^18^F-C2Am, with cell death markers (CC3, open bars, or TUNEL, closed bars) estimated from two sections (200 µm apart) from the same tumors, from both Colo205 and MDA-MB-231 models. (**b**) Pearson correlation analysis of tumor contrast, expressed as IA/g, % (left), SUV (middle) and T/b ratio (right), 1 h (top row) or 2 h (lower row) after ^18^F-C2Am administration, with tumor cell death (CC3, %). Tumor contrast is expressed as the signal ratio, post/pre-treatment. Pooled data (**a, b**) from Colo205 (*n* = 9, red symbols) and MDA-MB-231 (*n* = 9, blue symbols) tumor-bearing mice, treated with MEDI3039 (0.1 or 0.4 mg/kg, 24 h, i.v., filled symbols), 5FU (Colo205, 100 mg/kg, i.p., 24 h, open red symbols) or doxorubicin (MDA-MB-231, 100 mg/kg, i.p., 24 h, open blue symbols). Two-tailed, Pearson *P* and *R* values are shown (**a**). *CC3* cleaved caspase 3, *TUNEL* terminal deoxynucleotidyl transferase-mediated dUTP nick-end labeling. *T/b* tumor-to-blood ratio, *Tx* treatment

We analyzed several metrics of tumor contrast and their correlation with histological markers of tumor cell death in both the Colo205 and MDA-MB-231 tumor models (Fig. 7). IA/g (%), SUV, SUV_50_ and T/b were found to best correlate with these cell death markers in both tumor models (Fig. 7a), particularly with cleaved caspase-3 staining (CC3, open bars; *P* < 0.05; *R* > 0.6). SUV_90_ at 2 h post-probe administration correlated well with CC3 staining (Fig. 7a, *P* < 0.01, *R* > 0.6) but not with TUNEL (*P* > 0.05). Tumor contrast was also evaluated pre- and post-treatment for the same mice and expressed as ratios (post/pre-treatment) for IA/g (%), SUV or T/b (Fig. 7b). All three contrast metrics showed a good correlation with cleaved caspase-3 staining (CC3, %) in both tumor models, both at 1 h (*P* < 0.005; *R* > 0.62) and 2 h post-administration of ^18^F-C2Am (*P* < 0.03; *R* > 0.52). The slopes of the best fit lines for each metric, at 1 h or 2 h post-injection, were not significantly different. Overall, for all three metrics, a 20% increase in CC3 positivity resulted in a one unit increase in the post/pre-treatment tumor contrast. The correlation of ^18^F-C2Am tumor signal (expressed as %IA/g, SUV or T/b) post-treatment with CC3 positivity was in general better for the Colo205 model (0.69 < *R* < 0.86) than for the MDA-MB-231 model (0.47 < *R* < 0.68; Fig. S11). SUV_max_ showed a good correlation with CC3 positivity for MDA-MB-231 (*R* > 0.76) but not for Colo205 (*R* < 0.23; Additional file 1: Fig. S11). The correlation of the variation in ^18^F-C2Am tumor contrast with treatment (Additional file 1: Fig. S12) with CC3 positivity was better when expressed as ΔSUV (*R* = 0.694) than as ΔSUVmax (*R* = 0.567), 1 h after the injection of the contrast agent.

### ^18^F-C2Am signal in vivo and ex vivo versus histology

**Fig. 8:**
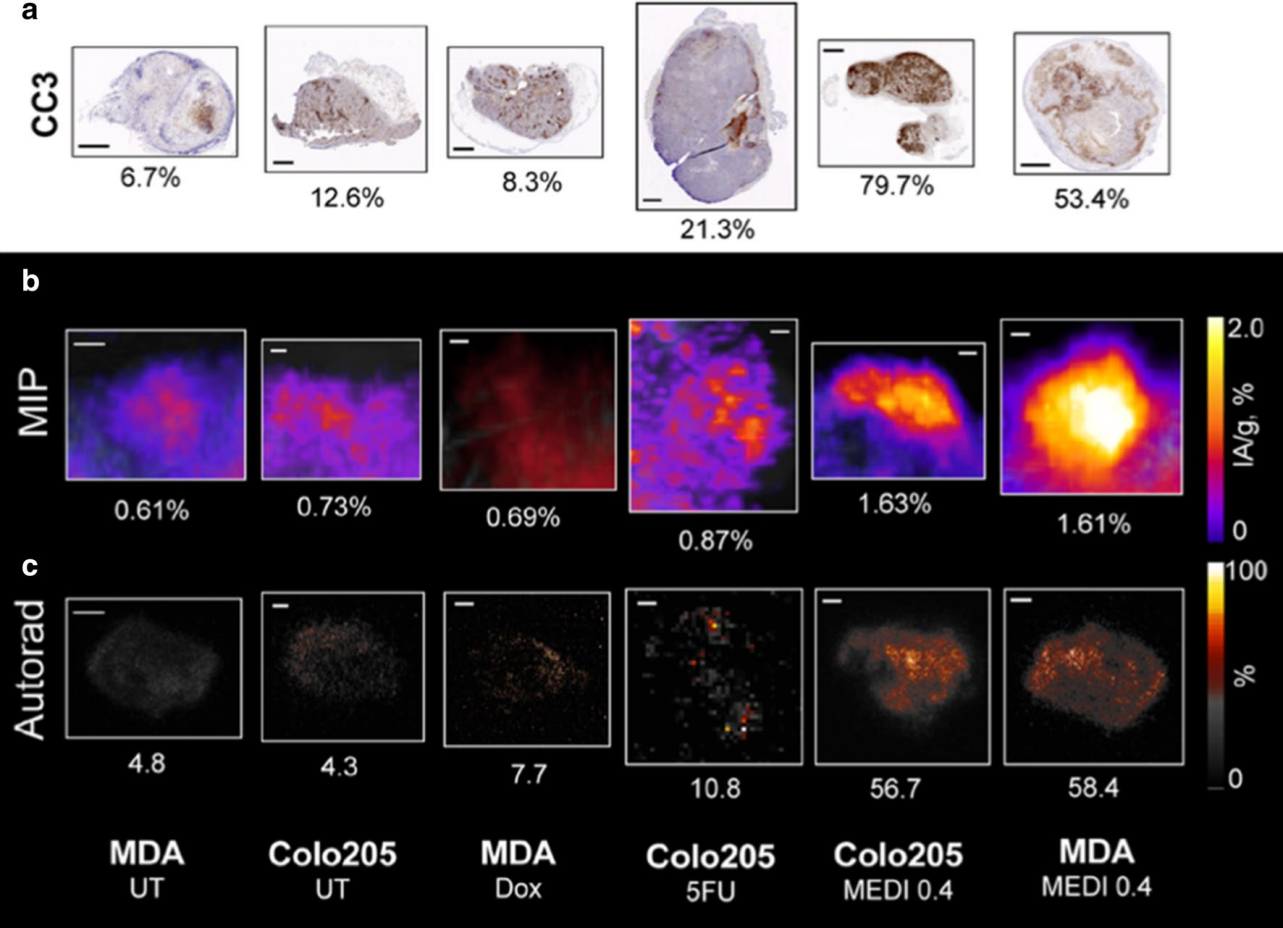
Correlation of ^18^F-C2A signal with immunohistochemical markers of cell death for representative MDA-MB-231 and Colo205 tumors. **a** Cleaved caspase-3 (CC3). Tumor section positive CC3 pixel count values (%) are shown. **b** Maximum intensity projection (MIP) of PET signal (IA/g, %). **c** Autoradiography of tumor sections acquired immediately after imaging (% of area with signal). Tumors were collected from different mice, bearing either Colo205 or MDA-MB-231, 2 h following administration of ^18^F-C2Am. MEDI3039 (MEDI) used at 0.4 mg/kg, i.v., for 24 h. 5FU and Doxorubicin (Dox) were both used at 100 mg/kg, i.p., for 24 h. *UT* untreated tumors

Next, we compared the percentage of the area occupied by dead cells on histology (CC3, %; Fig. 8a) with ^18^F-C2Am uptake in vivo (IA/g, %; Fig. 8b) and with the percentage of the area occupied by the ^18^F-C2Am signal in autoradiographs of excised tumor sections obtained following completion of the PET studies (Fig. 8c), 2 h post-administration of the imaging agent. Low levels of tumor cell death (CC3 < ∼10%, Fig. 8a) generated low levels of ^18^F-C2Am retention, as detected by PET in vivo (IA/g, ~ 0.7%, Fig. 8b) and autoradiography ex vivo (~ 6%; Fig. 8c). Tumor PET and autoradiography signals increased significantly (IA/g, ~ 1.7%, *P* < 0.05) with the extent of tumor cell death commonly generated by conventional therapies (CC3 > ~ 20%,) [24]. ^18^F-C2Am signal in tumors post-treatment was heterogeneous in both models, as shown in multi-slice images from the tumors (Additional file 1: Fig. S13), reflecting the heterogeneous distribution of cell death observed on histology (Fig. 8a).

### ^18^F-C2Am dosimetry

A dosimetry analysis was used to estimate the corresponding human dosimetry (Additional file 1: Table 1) using the interspecies scaling model proposed by Maina et al. [25] in order to examine the feasibility of translating ^18^F-C2Am into the clinic. ^18^F-C2Am cleared from the mice rapidly with 27.3 ± 6.1% and 113.9 ± 18% of the injected radioactivity collecting in the bladder at one and 2 h after injection, respectively (*n* = 6). The highest uptake was observed in the bladder, kidneys and liver. The estimated human-scaled effective dose was 12.5 ± 5.7 μSv/MBq (human effective dose estimate, in accordance with publication 103 of the International Commission on the Radiological Protection, ICRP [26]).

## Discussion

Imaging cell death can provide an indication of disease prognosis [27] and in cancer can be used to detect treatment response [3, 28]. Imaging agents that target various cell death-related events, such as cleaved caspase-3 (CC3) [24], changes in mitochondrial membrane potential [29], alterations in membrane permeability [30] and exposure of PS [31] and phosphatidylethanolamine (PE) [32] have been described [28], some of which have translated to the clinic [33]. However, the success of these agents has so far been limited. A CC3 targeted agent, ^18^F-ICMT-11, showed a lack of sensitivity for detecting cell death in breast and lung cancer patients treated with chemotherapy [34] and ^18^F-ML-10, an agent that detects changes in membrane permeability during apoptosis, failed to detect response to chemotherapy in a preclinical model of breast cancer [35]. Exposure of PS [31] and PE [32], which are externalized on the outer leaflet of the plasma membrane during apoptosis, or become accessible following plasma membrane disruption during necrosis, are attractive imaging targets since they provide a sustained and abundant target [14] that can give good image contrast [36]. ^99m^Tc-Annexin V, which binds PS, has translated to the clinic; however, it showed poor pharmacokinetics and non-specific binding [33]. Exposure of PE by dying cells has been exploited in the development of duramycin, a 19 amino acid tetracyclic peptide (~ 2 kDa), as a cell death imaging agent [37, 38]. However, despite its smaller size the clearance profile and contrast generated by ^99m^Tc-duramycin was similar to that observed here with ^18^F-C2Am. Following treatment of Colo205 tumors with a TRAILR2 agonist, the increase in tumor-to-muscle (T/m) contrast generated by ^99m^Tc-duramycin was 30.8 ± 1.9 ⨉ [37]. However, when corrected for non-specific retention using a control treatment antibody [37], the T/m was 6.9 ± 1.5×, which is similar to that observed here with ^18^F-C2Am (6.1 ± 2.1×). The retention of ^18^F-iC2Am, which is non-specific, was ~15%. More importantly, for clinical translation, ^18^F-C2Am showed comparable blood half-life (*t*_1/2_: 12.4 ± 2.2 min) to that reported recently for the small PET agent ^68^Ga-duramycin (*t*_1/2_: 17.3 ± 4.12 min) [39], but longer than that observed previously for the SPECT agent ^99m^Tc-duramycin (*t*_1/2_: 4.1 ± 0.3 min) [40]. Cell death-dependent contrast was obtained earlier using ^18^F-C2Am, at 2 h following agent administration, as compared to 4 h for ^99m^Tc-duramycin.

^18^F-C2Am also cleared more quickly than the γ-emitter labeled C2Am derivatives described previously (^99m^Tc-C2Am, *t*_1/2_: 582 ± 6 min; ^111^In-C2Am, t_1/2_: 480 ± 48 min) [15] and gave higher T/m ratios (∼ 6–10 ×) as compared to ^99m^Tc-C2Am (~ 4.3 ×) and ^111^In-C2Am (~ 2.2 ×), albeit with different tumor models (EL4 and Colo205) and treatment (chemotherapy) [15]. The C2Am fragment (< 3 kDa) detected in serum and urine, within 30 min of administration, is likely the result of proteolytic cleavage in the liver and/or kidney, which could contribute to this rapid clearance. The mean SUV_max_ for ^18^F-C2Am at 1 h post-administration (0.66 ± 0.18) was greater than that reported previously for a much larger ^18^F-labeled C2A derivative (^18^F-GST-C2A; 80 kDa; SUV_max_ = 0.47 ± 0.28) [10]. Moreover, no inactive C2A controls were performed in this previous study, suggesting that a substantial part of the tumor signal may have been caused by non-specific retention [10]. ^18^F-C2Am showed greater T/m contrast (two- to threefold) for identical levels of cell death and lower (2–10 ×) non-specific retention in other tissues post-treatment when compared to ^18^F-ICMT-11 [24], ^18^F-ML-10 [35], ^99m^Tc-Duramycin [36], and ^18^F-Annexin V [41].

Pre-blocking PS using unlabeled C2Am at tenfold greater molar dose than ^18^F-C2Am resulted in a ~ 70% reduction in tumor retention of ^18^F-C2Am. This reduction was much greater than expected given the high levels of PS predicted to be exposed by dying cells in these tumors (~ 30 μM), based on cell treatment experiments in vitro [14], and suggests that the PS accessible to C2Am in vivo is much less than this.

The levels of tumor cell death observed in the clinic can vary widely, from a few percent pre-treatment to 5–16% apoptosis following neoadjuvant treatment in breast cancer [42, 43]. ^18^F-C2Am detects both apoptosis and necrosis and therefore should be more sensitive than those agents that detect apoptosis alone, such as ^18^F-ML-10 [35]. Moreover, C2Am is also capable of binding PE [44], which like PS, is also externalized to the surface of dying cells.

The contrast observed here, where SUV doubled within an hour of ^18^F-C2Am injection following an increase in cell death from 8 to 20%, suggests that this agent should detect the expected levels of tumor cell death in the clinic. Analysis of ^18^F-C2Am signal heterogeneity, for example using Minkowski functionals, which we have used previously to analyze the heterogeneous distribution of a gadolinium chelate-conjugated derivative of C2A in magnetic resonance images [45], could further enhance the sensitivity of the agent for detecting treatment response.

The estimated human mean effective dose with ^18^F-C2Am is 12.5 ± 5.7 μSv/MBq, which was higher than that estimated for the SPECT tracer ^99m^Tc-duramycin (3.19 ± 2.16 μSv/MBq) [40], but similar to that observed recently for a small molecule PET tracer (^18^F-PSMA-11; 12.8 ± 0.6 μSv/MBq) [46], and for a biologic (^99m^Tc-Annexin-V; 9.7 ± 1.0 μSv/MBq) [47]. The total estimated effective human dose of ^18^F-C2Am equates to 5.6 ± 2.6 mSv (450 MBq injected), which is less than the PET element of a [^18^F]FDG PET/CT scan, 9.0 ± 1.6 mSv [48].

In conclusion, we have demonstrated ^18^F-C2Am as a PET agent for imaging cell death in vivo that showed fast renal clearance, good tumor contrast post-treatment and acceptable dosimetry. ^18^F-C2Am has the potential to be used in the clinic to assess early treatment response in tumors, such as breast, prostate, lung and colorectal.

## Supplementary Information

**Additional file 1**. Supporting methods and data.

**Additional file 2**. Supporting video 1.

**Additional file 3**. Supporting video 2.

## Data Availability

The datasets used and/or analyzed during the current study are available at 10.17863/CAM.60617.

## Abbreviations

BLI: Bioluminescence imaging
CC3: Cleaved-caspase-3 histological assay for apoptosis
C2Am: C2A domain of Synaptotagmin-I
PS: Phosphatidylserine
TRAILR2: (TNF)-related apoptosis-inducing ligand receptor-2
TUNEL: Terminal deoxynucleotidyl transferase dUTP nick-end labeling (histological assay for necrosis)
