# Supplementary material for: 18F-C2Am: a targeted imaging agent for detecting tumor cell death in vivo using positron emission tomography
Source: EJNMMI Res. 2020 Dec 9;10:151. doi: 10.1186/s13550-020-00738-7 (PMC7726082; doi:10.1186/s13550-020-00738-7)
Supplement: Supplementary file 1 — Additional file 1. Supporting methods and data. [file 13550_2020_738_MOESM1_ESM.docx]

**^18^F-C2Am : a targeted imaging agent for detecting tumor cell death *in vivo* using positron emission tomography**

Flaviu Bulat^1,2^, Friederike Hesse^1^, De-En Hu^1^, Susana Ros^1^, C. Willminton-Holmes^2^, Bangwen Xie^1^, Bala Attili^1^, Dmitry Soloviev^1^, Franklin Aigbirhio^3^, Finian. J. Leeper^2^, Kevin M. Brindle^1^, and André A. Neves^1^

^1^Cancer Research UK Cambridge Institute, University of Cambridge, Cambridge, CB2 0RE, United Kingdom;

^2^ Department of Chemistry, University of Cambridge, Cambridge, CB2 1EW, United Kingdom; and

^3^Wolfson Brain Imaging Centre, University of Cambridge, Cambridge, CB2 0QQ, United Kingdom

Corresponding author:

André A. Neves, CRUK Cambridge Institute, Li Ka Shing Centre, University of Cambridge, Robinson Way, Cambridge CB2 0RE, UK.

E-mail: [andre.neves@cruk.cam.ac.uk](mailto:andre.neves@cruk.cam.ac.uk)

**GENERAL EXPERIMENTAL PROCEDURES AND METHODS**

**Analytical size exclusion - HPLC method 1.** An XBridge BEH 125A (7.8 x 300 mm, 3.5 µm; Waters) size exclusion column, coupled to a UHPLC+ UltiMate 3000 HPLC system (Thermo Scientific Dionex) was used for the analysis of the [^18^F]FPenM prosthetic group and ^18^F-C2Am, following radiochemical synthesis (QC). A 100% PBS isocratic elution at 0.7 mL/min for 45 min, was used. 10% MeCN was added between 20 and 40 min.

**Analytical size exclusion - HPLC method 2.** A Superdex S75 increase 5/150 GL (5.0 x 155 mm, 9 µm; GE Healthcare) size exclusion column, coupled to a UHPLC^+^ UltiMate 3000 HPLC system (Thermo Scientific Dionex), was used for the analysis of mouse serum and urinary metabolites of ^18^F-C2Am. Isocratic elution, 100% PBS, 0.4 mL/min, 10 min run.

**Analytical RP-HPLC method 3.** A Primesphere C18-HC 110Å analytical column (250 x 4.6 mm, 5 µm; Phenomenex) was used for HPLC QC analysis of small organic ^18^F-labelled prosthetic groups using a 10 min 5-95% MeCN:H_2_O gradient, at 1 ml/min, followed by a 3 min step of 5:95 MeCN:H_2_O column re-equilibration wash.

**Radiotracer purification.** Semi-preparative purification of the radioactive prosthetic group was performed on a reverse-phase SUPELCOSIL LC-18 (250 mm x 10 mm; Sigma-Aldrich) column using a SYKAM S1122 dual piston solvent delivery system, which was remotely controlled by TRACERlab FXFN software.

**Nuclear magnetic resonance.** ^1^H NMR spectra were acquired at 400 MHz on a Bruker AV-400 Avance III. Chemical shifts (δ_H_) are quoted in parts per million (ppm), referenced to the appropriate residual solvent peak. ^13^C NMR spectra were acquired at 101 MHz on a Bruker AV-400 Avance III and chemical shifts (δ_C_) are quoted in parts per million (ppm), referenced to the appropriate residual solvent peak. ^19^F NMR proton coupled or decoupled spectra were acquired at 376 MHz on a Bruker AV-400 Avance III and chemical shifts (δ_F_) are quoted in parts per million (ppm). NMR data was analysed using TopSpin 4.0.6 version 1.0 or ACD Labs Academic Version.

**High-resolution mass spectrometry (HRMS).** Spectra were acquired on either a Waters Xevo G2-S QTOF or a Waters LCT Premier instruments. The first system uses a UPLC H-Class solvent management system running an 8 min 5-95% MeCN:H_2_O gradient at 0.2 mL/min with added 0.1% formic acid modifier) coupled to an electrospray ionisation (ESI) tandem mass spectrometer (MS/MS quadrupole and time-of-flight). The MS/MS is calibrated against a leucine-enkephalin internal standard, 1 mg/mL (m/z = 556.2771). MassLynx V4.1 software was used to run the samples, interpret the data and deconvolute protein spectra. This instrument was used for both organic small molecules and for proteins.

**Mass spectrometry.** Mass spectrometry **[1]** of protein samples was ran on a Waters Xevo SQD2 instrument to confirm the mass of the conjugate (*m/z* = 16408.5 [MH]^+^).

**Cell treatment.** Cell death was induced by addition of 1-10 pM of MEDI3039 to the culture medium, for 24 h at 37°C. An automated cell viability analyzer (Vi-Cell^®^, Beckman Coulter), based on the trypan blue dye assay, was used to monitor cell number and viability. Cell viability was also assessed in luciferase-expressing cells lines, by measuring luminescence using a PHERAstar FSX plate reader (BMG Labtech), 10 min after addition of D-luciferin solution (25 mg/mL; SynChem Inc.).

**Bioluminescence (BLI) imaging.** BLI was conducted 5 min following administration of D-luciferin (Perkin Elmer, 150 mg/kg, i.p. in PBS) using a Xenogen IVIS 200 camera (Perkin Elmer). BLI was typically performed prior to PET/CT imaging, under 2-3% isoflurane gas anesthesia mixed with 1:1 mix air/oxygen. Body temperature was maintained at 37°C. Regions of interest (ROI) were analyzed using Living Image software (v4.5, Perkin Elmer).

**Dynamic ^18^F-C2Am PET/CT imaging.** SUV (g/mL) was defined as C_img_/(IA/BW), where C_img_ is the activity concentration (MBq/mL) in the ROI, IA is the injected activity (MBq), and BW is the body weight of the animal (in grams). C_img-peak_ in SUV _peak_ was calculated from the (3⨉3⨉3) cube of voxels with the greatest mean of all similar cubes within the ROI. In SUV_peakM_, C_img-peakM_ was also calculated as for SUV_peak_, but the cube of voxels with the greatest mean also contained the pixel with maximum intensity [**2]**.

**Tissue ^18^F-C2Am autoradiography.** Tumor–bearing mice were injected (i.v.) with ^18^F-C2Am (∼3 MBq), culled after 120 minutes, tissues collected rapidly and immediately frozen by submersion in dry ice-cooled isopentane. Eight-micron thick axial cryosections were thaw-mounted onto coated glass slides (Cryostat, Leica). The slides were positioned with the tissue face placed onto a storage phosphor screen (GE Healthcare) overnight to produce autoradiographs that were scanned in phosphorescence mode using a flat-bed Typhoon scanner (GE Healthcare), at 10-μm resolution. The same slides were stained subsequently using hematoxylin and eosin (H&E), enabling manual co-registration of autoradiographic and H&E images.

**Immunohistochemistry.** Formalin-fixed paraffin-embedded tissue sections were stained for cleaved caspase-3 (CC3) and terminal deoxynucleotidyl transferase–mediated dUTP nick end labeling (TUNEL)***(3)***. A pixel count algorithm based on color and nuclear staining was developed using Aperio ImageScope software (vs. 12.3.3, Leica Biosystems)***(3)*** and used to calculate the percentage pixel positivity for each assay.

**Serum stability of ^18^F-C2Am *in vitro*.** Freshly labelled and purified [^18^F]-C2Am (1 MBq; 200 µL) was incubated in mouse plasma at 37℃ at a concentration similar to that generated *in vivo* immediately after administration (∼ 1 μM). Samples (10 µL) were collected over an 8 hour time course and analyzed by gel filtration HPLC (method 2; **Figure** S6)

**Serum and urine stability of ^18^F-C2Am *in vivo*.** Mouse whole blood and urine were collected at 15, 30, 60 and 120 minutes post injection of ^18^F-C2Am. Samples were centrifuged (3000 g, 4 °C, 5 min) and supernatants kept for HPLC analysis (method 2; **Figure** S7).

**Western blot analysis of TRAILR2 expression in cell lines.** Western blot analysis was used to measure TRAILR2 expression in three different human cell lines (Colo205, HT-29, MDA-MB-231), untreated and treated (1 pM, 5 pM, 10 pM MEDI3039; **Figure** S1). Cells were harvested, centrifuged (700 g, 5 min, 4°C), and resuspended in 350 μL RIPA buffer (ThermoFisher Scientific) containing complete mini EDTA-free protease inhibitor (Sigma Aldrich). Proteins were separated by SDS-Page and transferred to a nitrocellulose membrane using the iBlot 2 Dry Gel Transfer Device (ThermoFisher Scientific). Membranes were blocked with 1:1 Odyssey Blocking Buffer and Tris Buffered Saline (TBS) and incubated with an antibody solution (see Table A below) at 4°C overnight. Antibodies were detected using multiplexed IRDye secondary antibodies and membranes analysed using a Li-Cor Clx scanner (LI-COR Biosciences).

**Statistical analysis.** Data are shown as mean ± SD, unless stated otherwise. A two-tailed Student t-test was used for pairwise comparisons. Pearson *R* test was used to assess the significance of the correlation analysis. *P* values of <0.05 were considered significant. Statistical and graphical analysis were performed using GraphPad Prism (GraphPad Software, vs. 6.0).

**SYNTHETIC ORGANIC CHEMISTRY.** Furan protected maleimide **3** (Scheme 1) was synthesized in a moderate yield by adapting Coleman’s procedure **[5]**.

*N*-(5-fluoropentyl)maleimide **6** HPLC standard was synthesized to confirm the production of the equivalent radioactive molecule and to generate a concentration-absorbance calibration plot for determining molar radioactivity of the tracer.


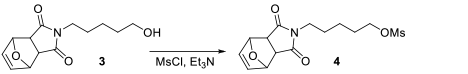


**Scheme S1.** Synthesis of *N*-(5-fluoropentyl)maleimide standard

**5-(1,3-Dioxo-1,3,3a,4,7,7a-hexahydro-2H-4,7-epoxyisoindol-2-yl)pentyl methanesulfonate.** 2-(5-Hydroxypentyl)-3a,4,7,7a-tetrahydro-1H-4,7-epoxyisoindole-1,3(2H)-dione (2.56 g, 10.187 mmol) was dissolved in dichloromethane (50 ml, Lot# 1417944, Fisher Scientific) in a nitrogen flushed round bottom flask. The solution was stirred in an ice bath for 10 min. Methanesulfonyl chloride ≥99.7% (946 μl, 12.225 mmol, 1.2 eq., Sigma-Aldrich) was injected dropwise under N_2_. Triethylamine ≥99.5% BioUltra (4.256 mL, 30.561 mmol, 3 eq., Sigma-Aldrich) was added dropwise to the stirred solution. The solution was allowed to warm to room temperature and stirred for 12 hours. The solvent was removed *in vacuo* and the yellow oil absorbed on Merck silica gel 60, loaded on a Redisep RF 25 g solid load cartridge and purified on a Teledyne Isco NextGen CombiFlash 300+ using a 40g HP Silica Gold column and an isocratic run with 100% EtOAc ≥99.8% for 10 column volumes with a flow of 110 mL/min. Fractions containing the product were collected and combined, solvent was removed *in vacuo* to yield the methanesulfonate precursor (2.698 g, 8.192 mmol, 80%) as a white crystalline powder.

R*_f_* (100% EtOAc) 0.65

m.p. = 94-96 °C

*δ_H_* (400 MHz, CDCl_3_): 1.40 (2H, m), 1.61 (2H, m), 1.76 (2H, m), 2.84 (2H, s), 3.00 (3H, s), 3.49 (2H, t, J = 7 Hz), 4.20 (2H, t, J = 6 Hz), 5.26 (2H, s), 6.51 (2H, s)

*δ_C_* (100 MHz, CDCl_3_): 22.5, 26.8, 28.5, 37.4, 38.4, 47.4, 69.6, 80.9, 136.5, 176.3

TOF-ESI-MS: found *m/z* = 330.1122 [MH]^+^([C_14_H_19_NO_6_S] + H^+^ requires 330.1011)

**
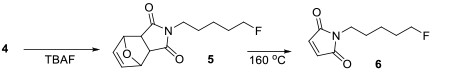
**

**Scheme S2.** Synthesis of precursor **6**

**1-(5-Fluoropentyl)-1H-pyrrole-2,5-dione.** Precursor **4** (1.34 g, 4.068 mmol) was dissolved in THF analytical grade (25 mL, Fisher Scientific) and added to a nitrogen flushed round bottomed flask. Tetrabutylammonium fluoride (1M, 6.1 mL, Acros Organics) was added under anhydrous conditions dropwise over 20 min while stirring at room temperature. The reaction was heated at reflux for 30 min, then cooled to room temperature and the solvent removed *in vacuo*. The resulting yellow oil was absorbed onto Merck silica gel 60. The resulting powder was dry loaded on a Redisep RF 25g solid load cartridge and purified on a Teledyne Isco NextGen CombiFlash Rf+ using an 80g Redisep Rf column and 20:80 Petroleum spirit 40-60:EtOAc gradient to 100% EtOAc over 5 column volumes. Fractions containing the product, were collected and combined, solvent was removed *in vacuo* to yield **5 (819 mg, 3.23 mmol, 79%) as a white solid.**

**Fluorinated compound 5 (**Scheme S2, **732 mg, 2.89 mmol) was dissolved in DMSO (≥99.6% extra dry, 5 mL,** Acros Organics**) in a nitrogen-flushed round bottomed flask** and heated to 160 °C for 30 min with periodic monitoring of reaction progression by thin-layer chromatography (in 100% EtOAc). Upon completion, the flask was cooled by submersion in an ice/water bath and Merck silica gel 60 added and the resulting powder was dry loaded onto a Redisep RF 25 g solid load cartridge and purified on a Teledyne Isco NextGen CombiFlash Rf+ using a 40 g Redisep Rf column and an isocratic run with 100% Et_2_O puriss ≥99.8% (Honeywell) for 8 column volumes with a flow rate of 30 mL/min. Fractions containing the product, were collected and combined, solvent was removed *in vacuo* to yield **6** (338.4 mg, 1.827 mmol, 63%) as a colorless oil.

R*_f_* (100% EtOAc) 0.92

*δ_H_* (400 MHz, CDCl_3_): 1.39 (2H, m), 1.68 (4H, m), 3.53 (2H, t, J = 7 Hz), 4.42 (2H, dt, J = 47 and 6 Hz), 6.68 (2H, s)

*δ_C_* (100 MHz, CDCl_3_): 22.4 (d, J = 6 Hz), 28.1, 29.8 (d, J = 20 Hz) 37.6, 83.7 (d, J = 165 Hz), 134.1, 170.8

*δ_F_* (376 MHz, CDCl_3_): -218.6 (tt, J = 47 and 25 Hz)

TOF-ESI-MS: found *m/z* = 186.2220 [MH]^+^ ([C_9_H_12_FNO_2_]+H^+^ requires 186.0930)

LC-MS: *m/z* = 186.2 [MH]^+^ detected at 1.49 min.

**Synthesis of FPenM-C2Am HPLC standard.** C2Am was reduced with 0.4 mL solution of HNE buffer (20 mM HEPES, 100 mM NaCl, 5 mM EDTA, pH 7.4) and DTT (10 mM) for 30 min at room temperature. DTT was removed by spin filtration and C2Am was washed with HNE buffer three times. Freshly reduced C2Am (1.29 mM) was added to **6** (9 μM, ~1000 eq.) in PBS to yield a FPenM-C2Am conjugate standard for HPLC analysis. Conjugated protein was purified by spin filtration (Amicon Ultra 0.5 mL 3 kDa centrifugal filter cartridge) and concentrated to yield pure FPenM-C2Am (1.29 mM). Mass spectrometry confirmed the mass of the conjugate (*m/z* = 16408.5 [MH]^+^).

**Radiochemistry.** A one-pot, two-step (Scheme 1) automated synthesis of *N*-(5-[^18^F]fluoropentyl)-maleimide ([^18^F]FPenM) was developed on a GE TRACERLab FX_FN_ automated module. The radiosynthesis, based on a modified version of the methods described by Fujita *et al.* **[6]**, purification and formulation took 58±5.8 min (*n* = 12)

Scheme S3. ^18^F-labelling of precursor 4

TRACERlab FX_FN_ automated synthesis. Cyclotron water (~2.4 mL) containing up to 30 GBq H[^18^F]F was loaded on a Waters Sep-Pak Accell Plus QMA Carbonate light cartridge. Kryptofix-222 solution (1.5 mL solution containing 2.5 mg K_2_CO_3_, 15 mg Kryptofix-222, 95:5 MeCN:water) was used to elute K[^18^F]F-K_222_ from QMA cartridges into the reactor vessel. The GE Healthcare TRACERlab FX_FN_ automated synthesizer was coupled to an external UV diode array detector set at 220 nm. Radioactive tracer was purified by semi-preparative HPLC as described above.

TRACERlab FX_FN_ module vial loading and setup.

Cartridge #1 QMA – Waters Sep-Pak Accell Plus QMA Carbonate Plus Light cartridge

Cartridge #2 Intermediate – Waters Sep-Pak Alumina N Plus light cartridge

Cartridge #3 SPE – Waters Sep-Pak Plus Light C18 cartridge

Vial 1 – 1.5 mL Kryptofix-222 elution solution (2.5 mg K_2_CO_3_, 15 mg Kryptofix K_222_, 1.5 mL 95:5 (v/v) MeCN: H_2_O)

Vial 2 – MeCN (2 mL) dried over molecular sieves

Vial 3 – Mesylate precursor **4** (5 mg, 15.2 µmol, 15.2 mM) in dry DMSO (1 mL)

Vial 4 – Methanesulfonic acid (20 µL) in dry DMSO (0.5 mL)

Vial 5 – DMSO (0.5 mL), NH_4_OAc buffer (150 mM, 1 mL, pH 6.8) + ascorbic acid (2 mg/mL) + AcOH (10 µL), final buffer pH 4.12

HPLC Vial – NH_4_OAc buffer (150 mM, 2 mL, pH 6.8) + ascorbic acid (2 mg/mL) and AcOH (20 µL), final buffer pH 4.12

Vial 6 – None

Vial 7 – None

Vial 8 – EtOH (0.3 mL)

Vial 9 – HBS buffer (1 mL, pH 7.4)

Dilution Flask – NH_4_OAc buffer (50 mM, 30 mL, pH 6.8) + ascorbic acid (1 mg/mL) +

AcOH (300 µL), final buffer pH 3.65

Product vial – HBS buffer (100 µL, pH 7.4) + ascorbic acid (1 mg/mL), final buffer pH 4.5

HPLC Solvent – 38:62 MeCN (183 mL): NH_4_OAc buffer (50 mM, 30 mL, pH 6.8) + ascorbic acid (0.1 mg/mL) + AcOH (300 µL), final buffer pH 5.6-5.7

Automated radiochemical synthesis of [^18^F]FPenM. Using a GE TRACERlab FX_FN_, precursor 4 (5 mg, 15.2 µmol) in dry DMSO (1 mL) was added to dried K[^18^F]F-Kryptofix-222 (27±1 GBq at start-of-synthesis).

The mixture was stirred at 92 °C for 12 min then methanesulfonic acid (20 µL) in dry DMSO (0.5 mL) was added and heated at 160 °C for 6 min. The reaction mixture was cooled to < 35 °C and passed through a pre-conditioned (DMSO, 2 mL) Waters Sep-Pak Alumina N Plus light cartridge. The collecting vial was pre-filled with NH_4_OAc solution (22 mg NH_4_OAc, 2 mL H_2_O) with ascorbic acid (4 mg) and acetic acid (20 µL). The reactor was washed with 0.5 mL DMSO and NH_4_OAc solution (11 mg NH_4_OAc, 1 mL H_2_O, 150 mM) with ascorbic acid (2 mg) and AcOH (10 µL). The prosthetic tracer (5 mL) was then loaded on the HPLC column and eluted with 38% MeCN in 50 mM NH_4_OAc (with 1% ascorbic acid and 1% AcOH). The purified peak at around 14 min (see Figure S2b) was collected and diluted in 30 mL NH_4_OAc buffer (50 mM NH_4_OAc, ascorbic acid 1 mg/mL, acetic acid 1% v/v, pH 5.6). The solution was flowed through an ethanol/water (2 mL/10 mL) pre-activated Sep-Pak Plus Light C18 cartridge and the N-(5-[^18^F]fluoropentyl)maleimide ([^18^F]FPenM) eluted with ethanol (0.3 mL) and 1 mL HEPES buffered saline (10 mmol/L HEPES, 150 mmol/L NaCl, pH 7.4) to give ~ 2 GBq (end-of-synthesis) in a volume of 1.3 mL. This was produced in 58±5.8 min (*n* = 12) in moderate radiochemical yield (12±3%, decay corrected to start-of-synthesis, n = 11) and high radiochemical purity (95±3.4% by radio-HPLC, n = 11). Specific (1144±161 GBq/mg (*n* = 3)) and molar activities (212±30 GBq/µmol (*n* = 3)) of [^18^F]FPenM were determined by UV-HPLC monitoring at 220 nm.

#### *N*-(5-[^18^F]fluoropentyl)maleimide-C2Am ****(^18^F-C2Am)**** ****bioconjugation.**** C2Am (123 µg, 25.6 µl, 7.58 nmol, 297 µM) was conjugated to 8 (150 µL, 359 MBq) at RT, 30 min, in HPS buffer, to yield, after gel filtration, *N*-(5-[^18^F]fluoropentyl)maleimide-C2Am (56.1 µg, 3.42 nmol) with a conjugation yield of 87±4.3% (*n* = 5) by radio-HPLC. The *N*-(5-[^18^F]fluoropentyl)maleimide-C2Am solution (175.6 µL, 42.7 µM) was purified on a Sephadex G-25 desalting cartridge (PD-10, Illustra NAP-5 GE Healthcare Life Sciences) and collected in 3 fractions. The 1^st^ fraction eluted with PBS (300 µl) was discarded. The 2^nd^ fraction (~100 MBq end-of-purification, 56.1 µg, 3.42 nmol, 550 µl, 5.7 µM) was eluted with PBS (550 µl) to yield >99% pure (by radio HPLC) [^18^F]FPenM-C2Am. The molar activity of [^18^F]FPenM-C2Am was 29.5±10.6 GBq/µmol ( *n* = 5) (1.8±0.65 MBq/µg) at the end-of-synthesis (conjugation and purification) and ~16.6 GBq/ µmol (~1 MBq/µg) at the time of injection. A Merck Direct Detect Infrared spectrometer was used to determine protein concentration.

**SUPPLEMENTARY DATA**

**^1^H-NMR and HRMS spectra**


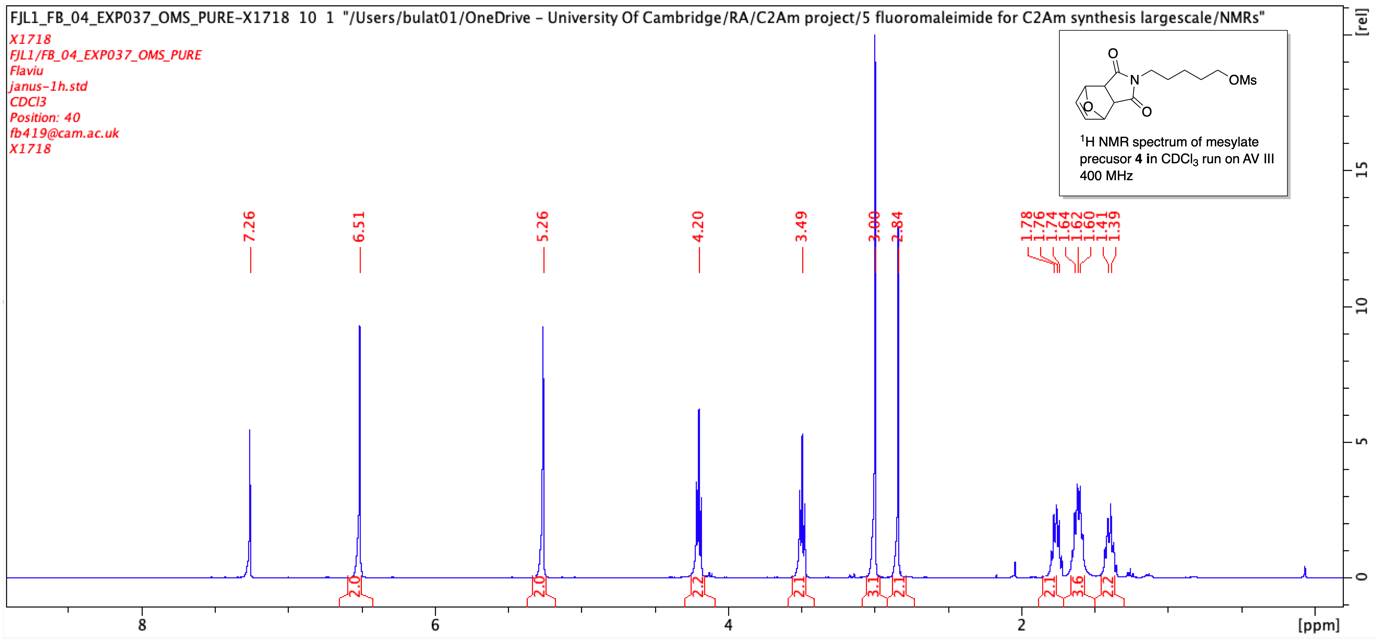


**
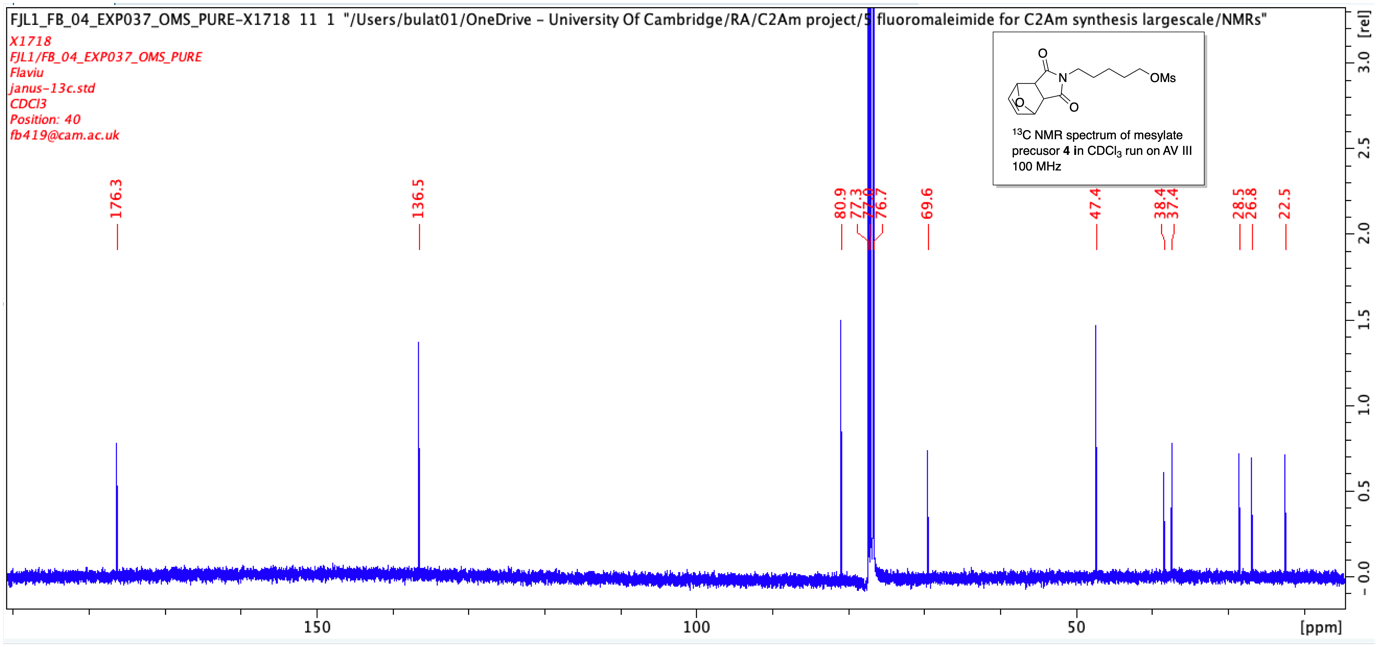
**


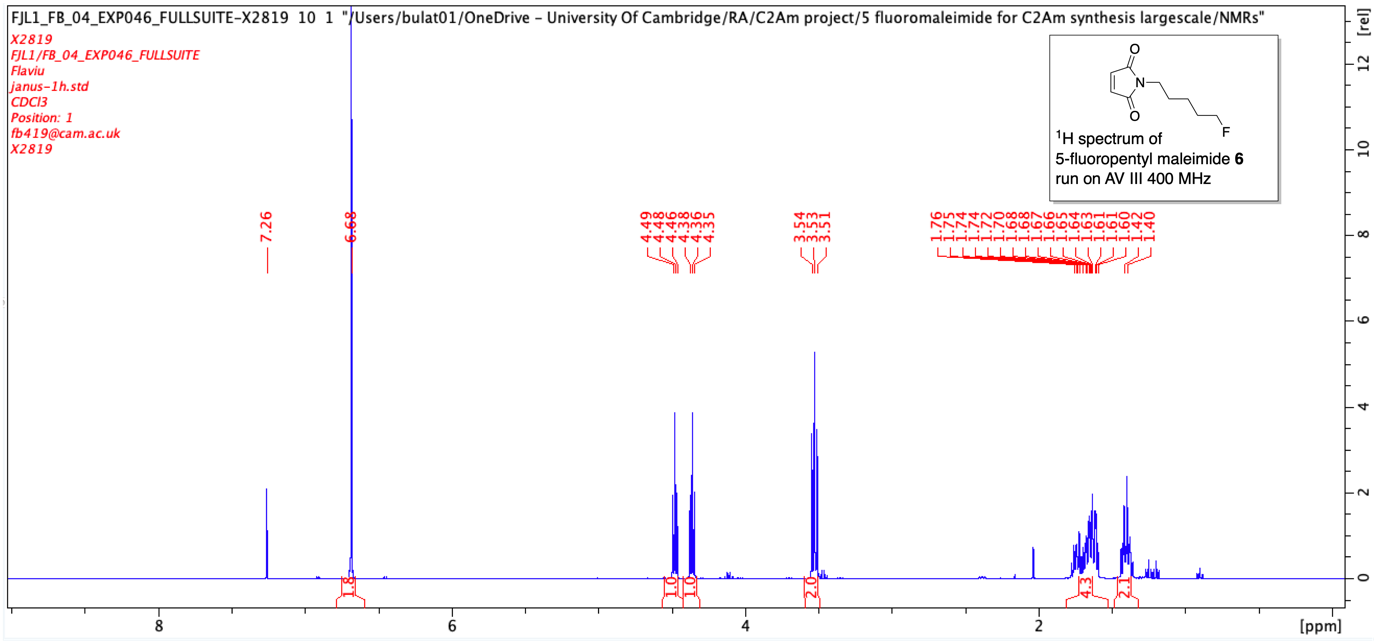


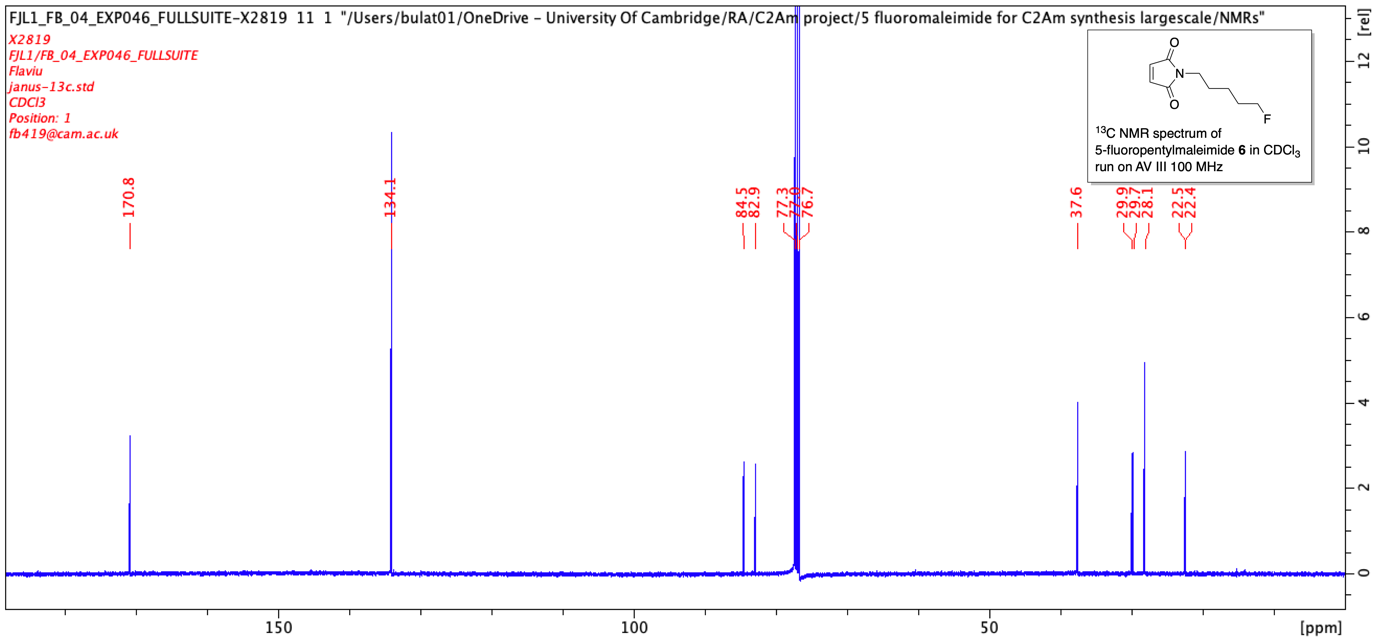


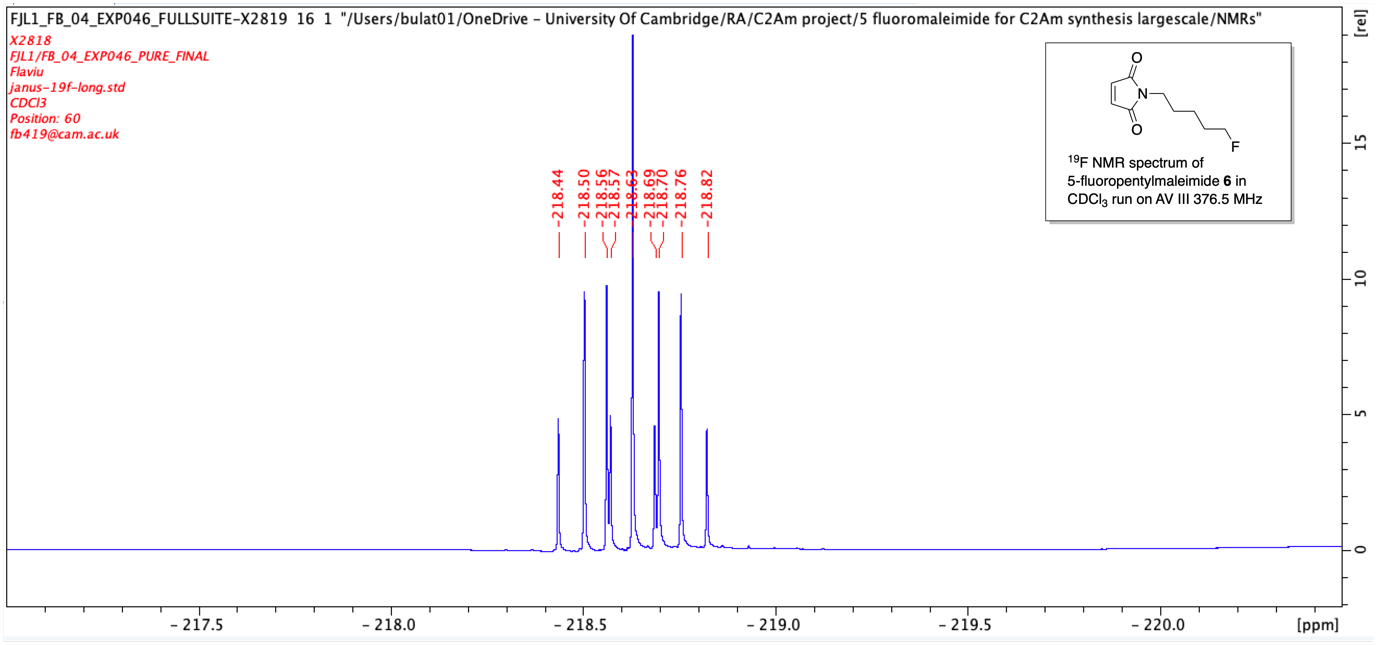


**^1^H-NMR and HRMS spectra cont.**


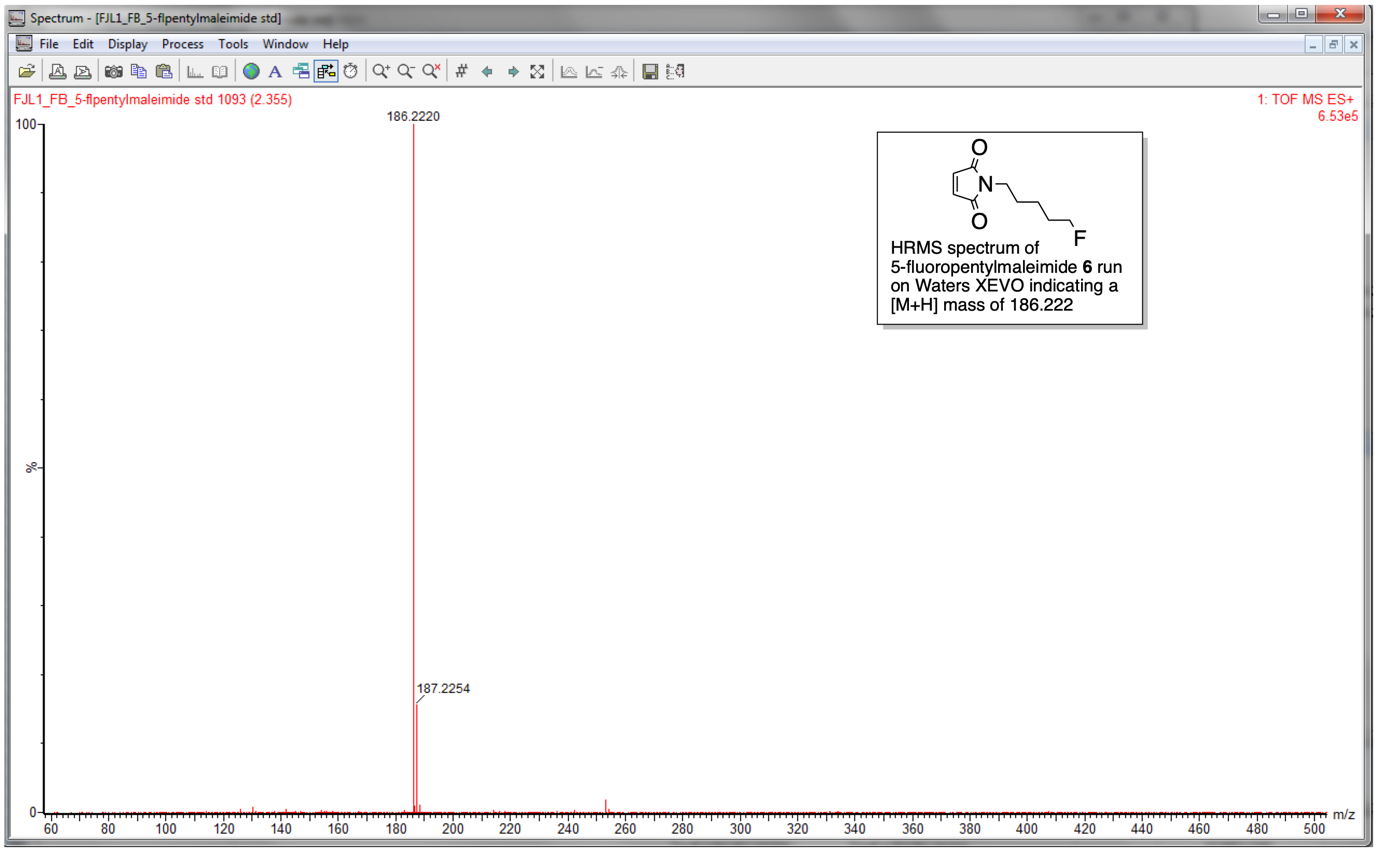


**^1^H-NMR and HRMS spectra cont.**


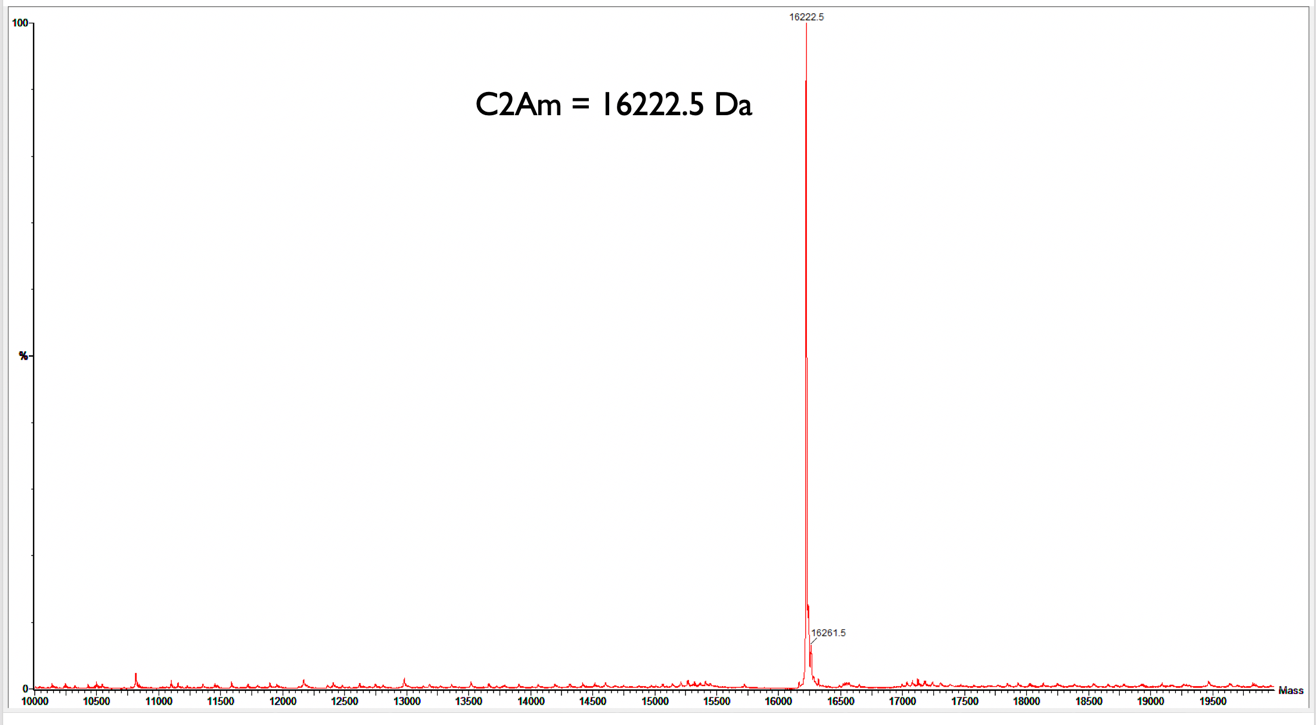


A


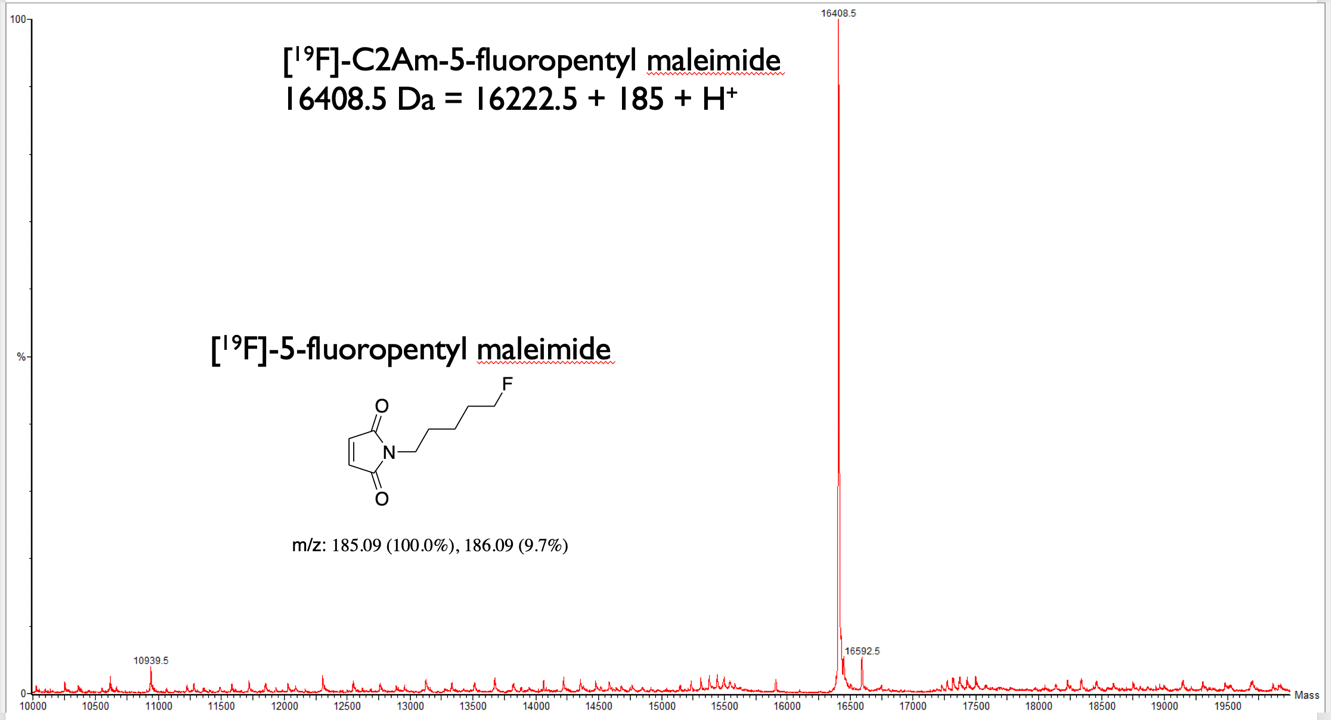


B

**High resolution ESI MS spectra of unlabeled (A) C2Am and (B) ^19^F-C2Am**

**Figure S1 –** (A) Three-step synthesis of precursor 4. (B) Synthetic route to N-(5-fluoropentyl)maleimide 6.

**Figure S2A –** **Validation of [^18^F]FPenM radiosynthesis.**


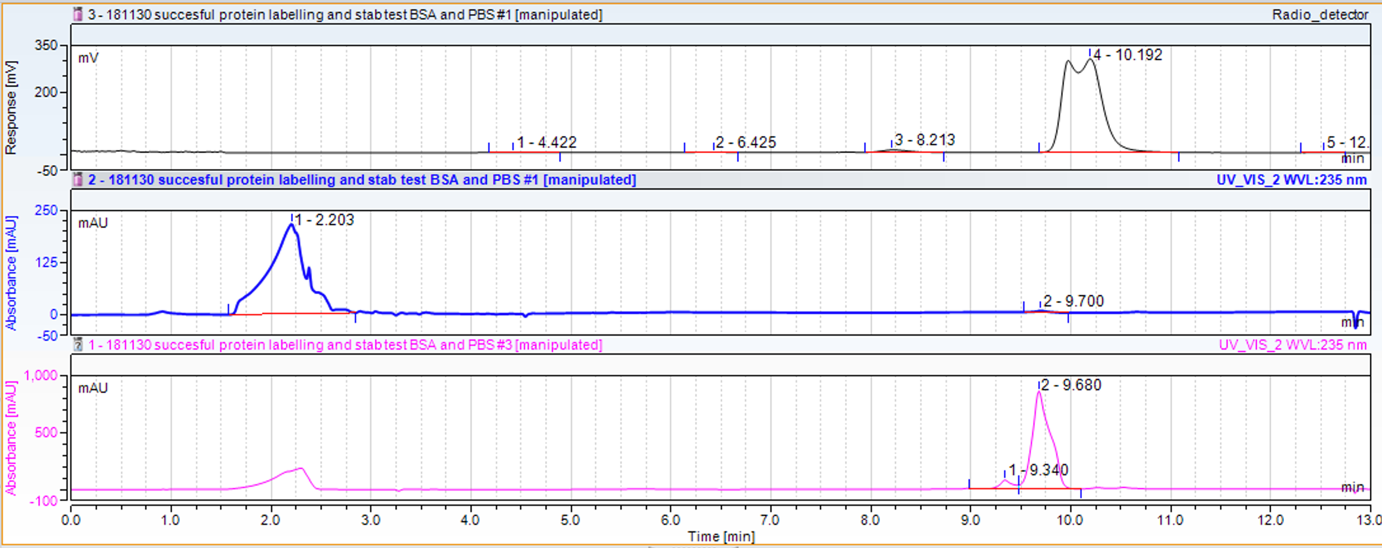


Top radiochromatogram of analytical HPLC analysis showing the radio-peak 4 at 10.2 min from **[^18^F]FPenM.** UV chromatogram (middle) shows peak 2 at 9.7 min from FPenM. UV chromatogram (bottom) shows the **spiked sample with a FPenM HPLC standard added (peak 2 at 9.7 min). Analytical reverse-phase (HPLC method 3) was used.**

**Figure S2B –** Semi-preparative purification of [^18^F]FPenM.

**Figure S3- Labelling of C2Am with ^18^F**


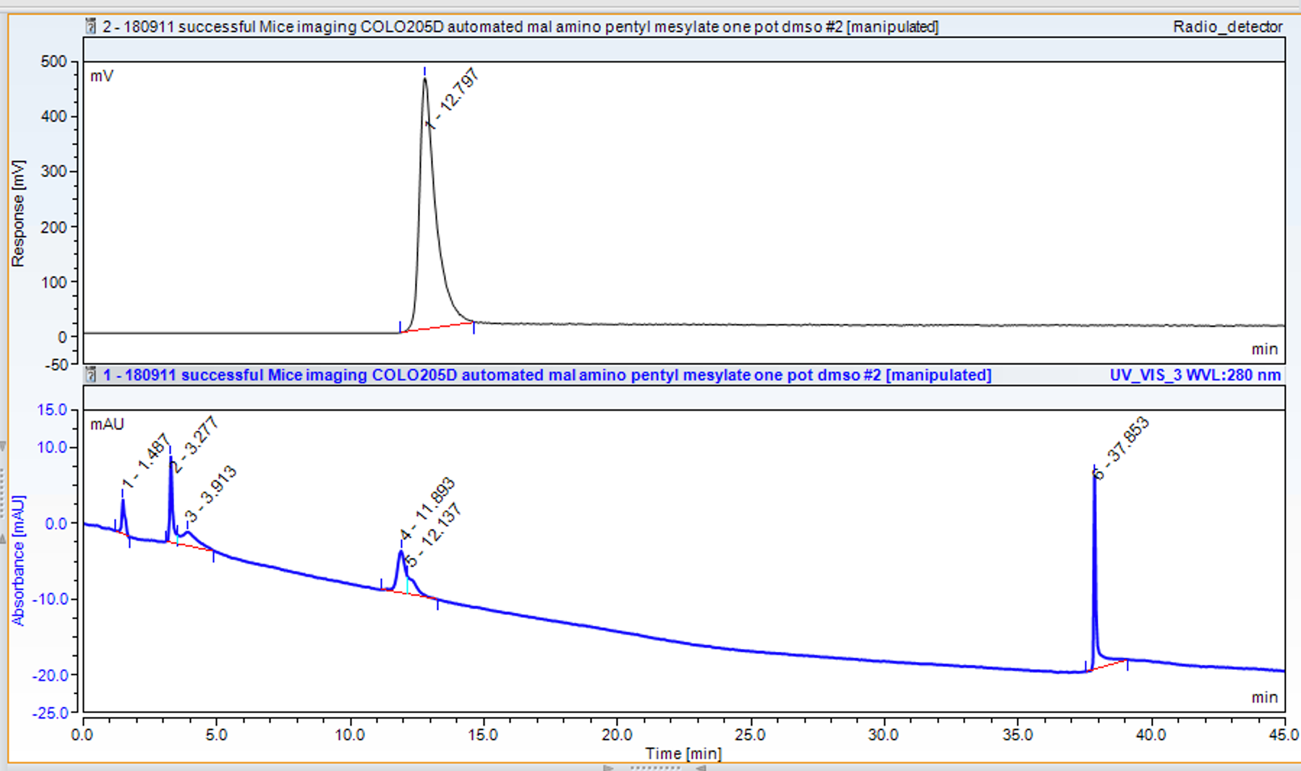


Chromatogram of pure [^18^F]FPenM-C2Am (^18^F-C2Am). Top chromatogram is the radiodetector trace, showing [^18^F]FPenM-C2Am elution at 12.8 min. Lower chromatogram shows the UV (280 nm) trace, showing C2Am at 11.9 min (FPenM-C2Am). Peaks 1, 2 and 3 are probably caused by dimerization of unlabeled C2Am (disulfide bond formation) and aggregation of the dimers. Peak 6 (UV trace) is likely to be ascorbic acid. Analytical size exclusion – (HPLC method 1 was used).

**Table A-** Antibodies used for Western blotting

| **Antibody Target** | **Primary Antibody** | **Manufacturer** | **Secondary Antibody**  **and dilution** |
| --- | --- | --- | --- |
| **DR5** | Rabbit polyclonal IgG | Cell Signaling | Goat anti-rabbit 1:5000 |
| **GAPDH** | Mouse mAb | Sigma-Aldrich | Goat anti-mouse 1:15000 |

**Table 1 - Estimated human dosimetry profile of ^18^F-C2Am.**

**
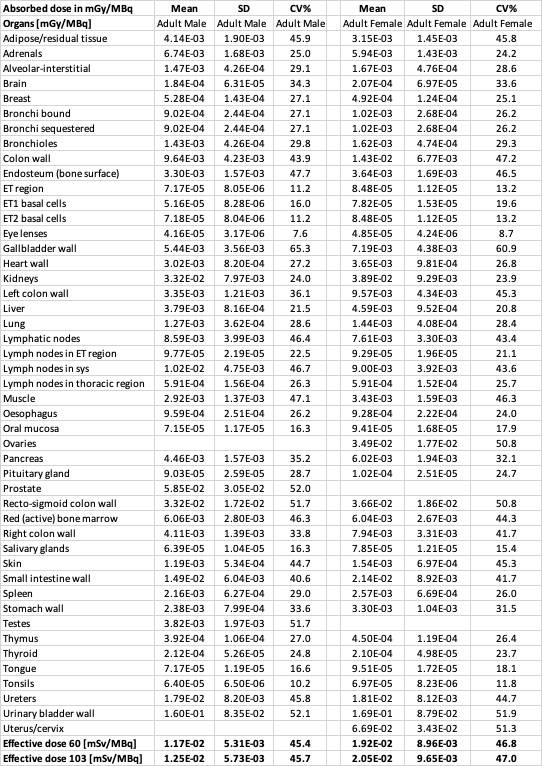
**

Data shown as mean ± SD of mean ( *n* = 6). Human dosimetry estimated from dynamic mouse PET data, based on the method proposed by Maina et al. **[7]** and using IDAC 2.1 software [**8**]

**Figure S4 – Western blot analysis of TRAILR2 (DR5) expression in cell lines.**

**
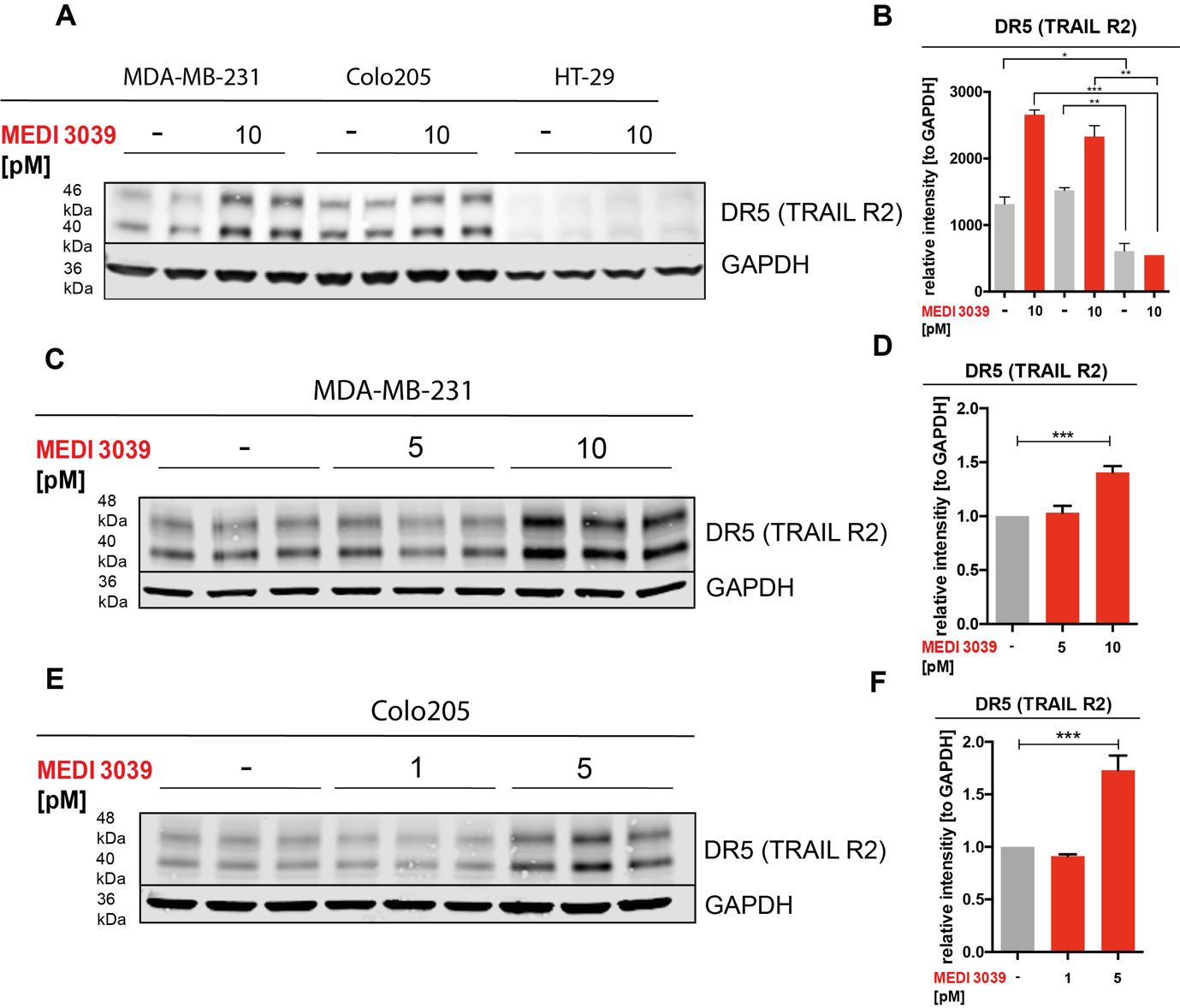
**

The expression levels of DR5 were evaluated in Colo205 (A,B, E, F) and MDA-MB-231 (A-D) cells, treated with MEDI3039. HT-29 cells (A,B), which do not express the receptor were used as a negative control. Expression levels increased with MEDI3039 treatment in both Colo205 and MDA-MB-231 cells. The effect was more pronounced in Colo205, particularly at low concentration (5 µM). (B, D, F) mean ± SD, *n* = 3, * = *P* <0.05, ** = *P* <0.005, *** = *P* <0.001

**Figure S5 – Flow cytometric analysis of cell death using C2Am-AF750 and Annexin-V-750**

**
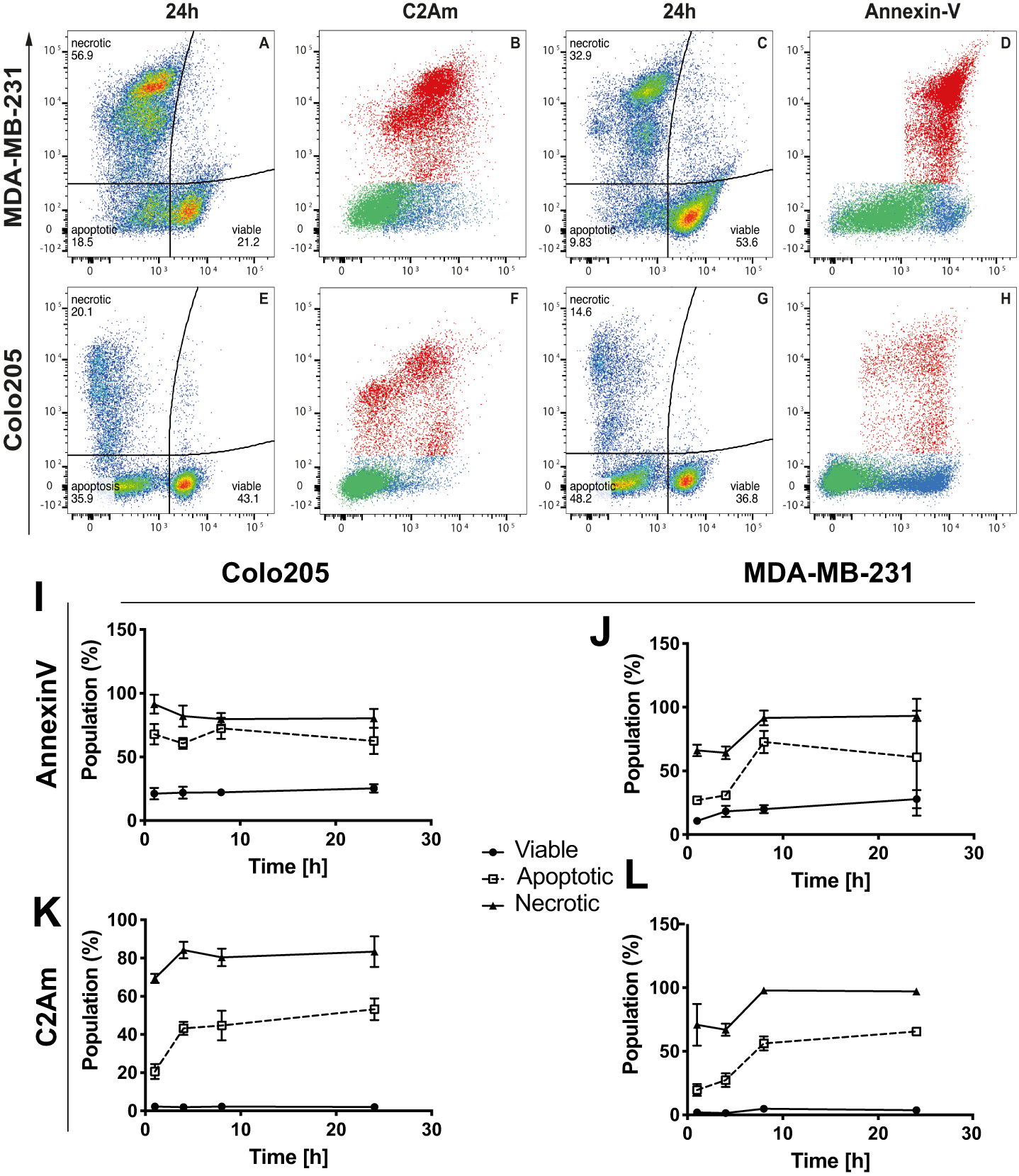
**

Flow cytometric analysis of MEDI3039-treated MDA-MB-231 and Colo205 cells shows three distinct populations (top 8 plots), which were gated based on their levels of UV_A_ autofluorescence (NADH content, x-axis) and plasma membrane integrity (Sytox R, y-axis; plots **A, C, E, G**) as: viable (green), apoptotic (blue) and necrotic (red) cells. C2Am-AF750 (C2Am) or Annexin-V (Annexin-V), near infra-red fluorophore-labelled derivatives of the two proteins (plots **B, D, F, H**) preferentially bound apoptotic and necrotic cells, in comparison with viable cells. % of each cell population that labelled with either Annexin-V (**I, J**) or C2Am (**K, L**), are shown for MDA-MB-231 and Colo205 cells, following treatment with MEDI3039 (mean ± SD, *n* = 3), error bars lie within the symbols when not shown.

**Figure S6 – Histopathological assessment of cell death in mouse tissues.**

Cell death was evaluated in mouse spleen, liver and tumor using CC3 and TUNEL assays. Tissues were collected from untreated and MEDI3039-treated (0.4, 0.8 mg/kg, 24h, i.v.). *n* = 3 per group, **** = *P* <0.0001

**Figure S7** – **Stability of ^18^F-C2Am in serum *in vitro*.**

**
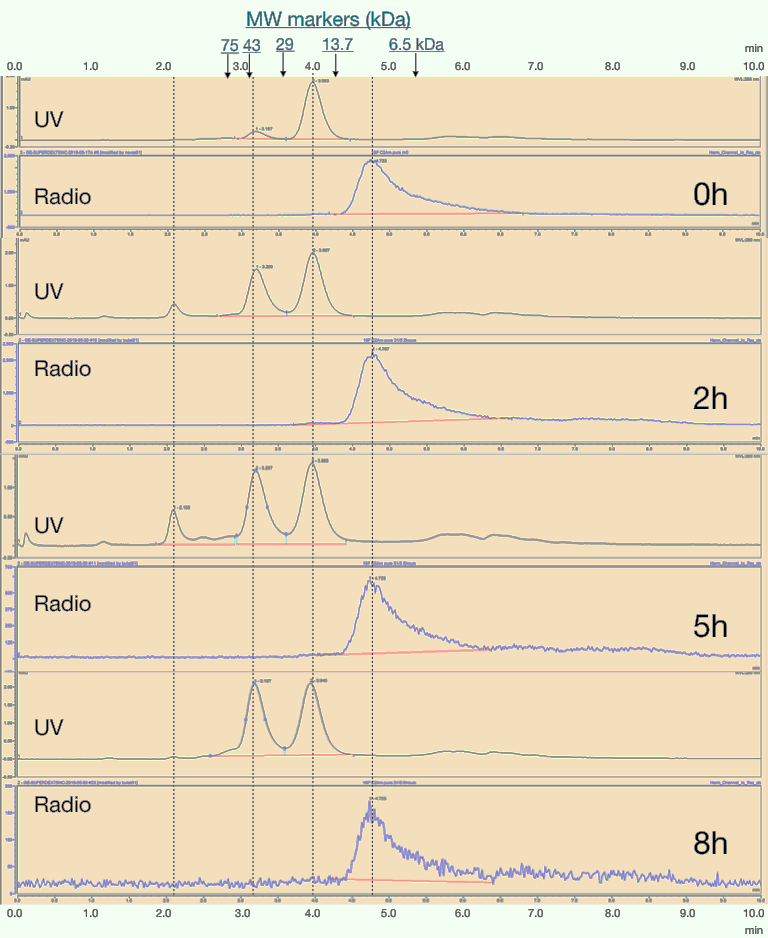
**

Purified ^18^F-C2Am was incubated in mouse serum at 37 ℃ for up to 8 h and then analyzed by gel filtration HPLC. Chromatograms show first the UV absorption (280 nm) trace, second the radioactivity trace (Radio). ^18^F-C2Am elutes at ∼4 min (UV) and ∼4.8 min (radio). A larger molecular weight species was detected at ∼3 min (estimated MW∼35 kDa). The latter results from dimerization of unlabeled C2Am. The gel filtration column used (Superdex S75 increase 5/150), which has a fractionation range of 3-70 kDa, was ran separately using small globular proteins (Cytiva LMW kit) as molecular weight markers (arrows top). (HPLC method 2 was used).

**Figure S8 -** ^18^F-C2Am metabolite analysis in serum (A) and urine (B) from a MEDI3039-treated mouse following injection of ∼5 MBq (5 μg) of ^18^F-C2Am and in serum (C) and urine (D) following injection of ∼15 MBq (15 μg) of ^18^F-C2Am.

**
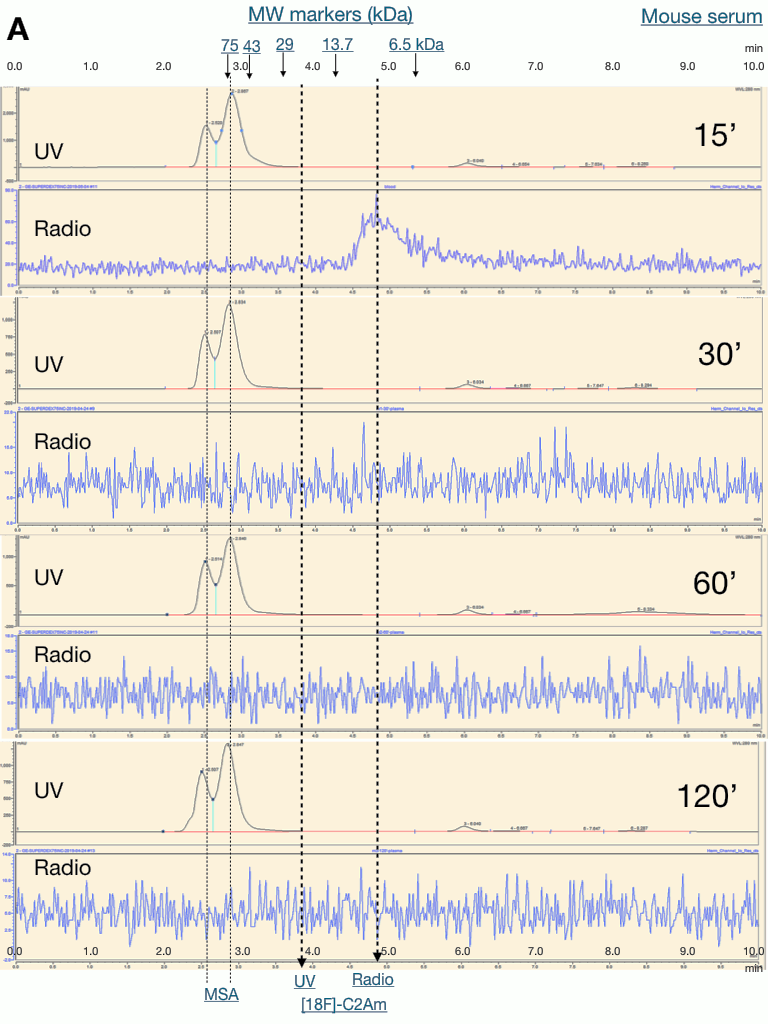
**

**S8A.** The major protein present was, as expected, MSA (mouse serum albumin), which was not radiolabeled (MWs ∼60 and ∼120 kDa, corresponding to peaks in (A) at retention times of ∼2.5 and ∼2.8 min). Intact ^18^F-C2Am (∼4.8 min in Radio channel) was visible at 15 min post injection but could not be detected after that due its short blood half-life (11 min). The gel filtration column used has a fractionation range of 3-70 kDa. Molecular weight markers are shown (arrows on top). (HPLC method 2 was used).

**
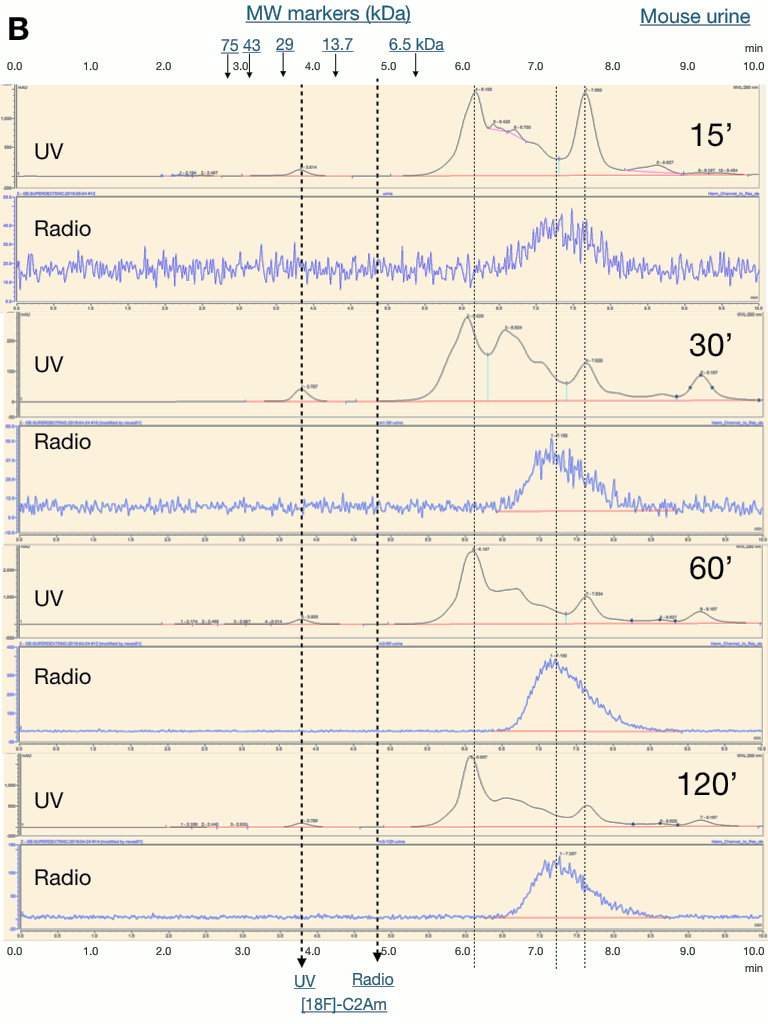
**

**S8B.** **^18^F-C2Am metabolite analysis in urine from a MEDI3039-treated mouse following injection of ∼5 MBq (5 μg) of ^18^F-C2Am.** Intact ^18^F-C2Am and various small molecular weight metabolites, could be detected in urine from 15-120 min post injection, indicating renal metabolism and excretion of the imaging agent. The main radiolabeled metabolite (∼7.3 min) corresponds to an estimated MW < 3 kDa. The gel filtration column used has a fractionation range of 3-70 kDa. Molecular weight markers are shown (arrows on top). (HPLC method 2 was used).

**S8C.** **Data shown corresponds to HPLC analysis of mouse serum and urine collected 30 minutes following iv injection of the agent**. A larger activity of ^18^F-C2Am was injected in order to follow the metabolism of the agent for a longer period of time. Blue arrow heads indicate a ^18^F-C2Am metabolite. The gel filtration column used has a fractionation range of 3 -70 kDa. Molecular weight markers are shown (arrows on top). Note that the prosthetic group (5-fluoropentylmaleimido) was analyzed ca. 2h post synthesis; the chromatograms show two peaks (at **∼** 8 and 8.5 min on UV), which correspond to the intact and oxidized forms of the compound. MSA-mouse serum albumin (HPLC method 2 was used)

**S8D.** **Data shown corresponds to HPLC analysis of mouse serum collected 60 minutes following iv injection of the agent.** A larger activity of ^18^F-C2Am was injected (∼15 MBq or 15 μg of ^18^F-C2Am), in order to follow the metabolism of the agent for a longer period of time. Blue arrow heads indicate a ^18^F-C2Am metabolite. The gel filtration column used has a fractionation range of 3-70 kDa. Molecular weight markers are shown (arrows on top). MSA-mouse serum albumin (HPLC method 2 was used).

Note that the retention times shown in C and D are longer that those shown in A, and B, which is due to a longer HPLC fluidics path used for the experiments shown in C and D.

**Figure S9 – ^18^F-C2Am** **tumor signal, 24 h post treatment, expressed as SUV and SUV_max_.** Dark and light bars indicate signal 1h, and 2h post injection. Data shown in red and blue correspond to Colo205 and MDA-MB-231 models, respectively. Average values are shown for each model at 1h (<1h>) and 2h (<2h>) post injection, and for the pooled data (top left inside charts). n=9, Colo205, red; n=9, MDA-MB-231, blue.


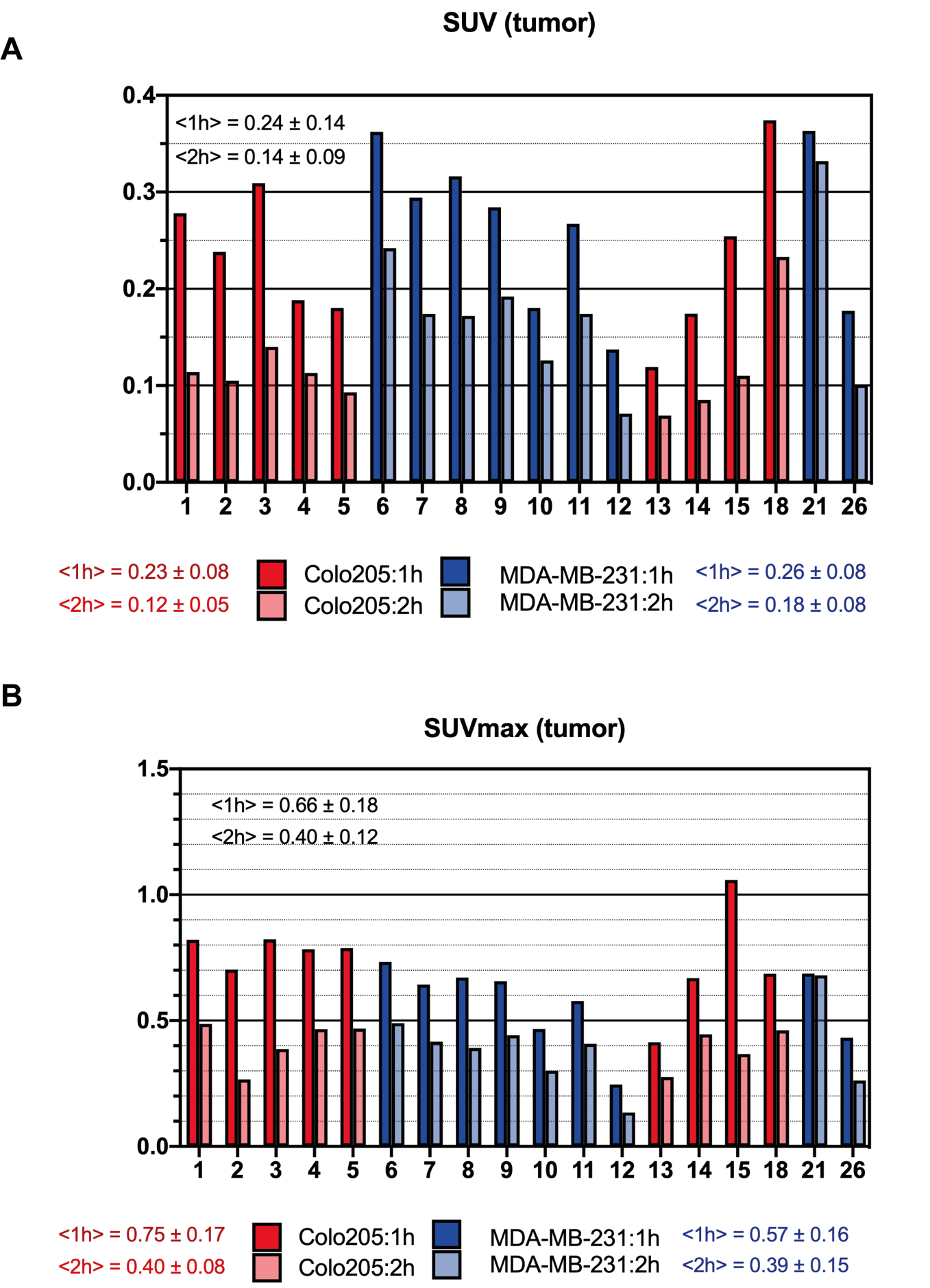


**Figure S10 – Dose dependent accumulation of ^18^F-C2Am** **in** **tumors post treatment.** Tumor data was acquired before (PRE) and 24h post (POST) treatment with MEDI3039 (at either 0.1, 0.2 or 0.4 mg/kg) and 2h post injection of ^18^F-C2Am. Data (IA/g, %) was standardized to pre-treatment tumor signal. Symbol legend: circles, MDA-MB-231 model; triangles, Colo205 model. Colors: transparent, 0.4; blue: 0.1, pink, 0.2 mg/kg MEDI3039 dose.


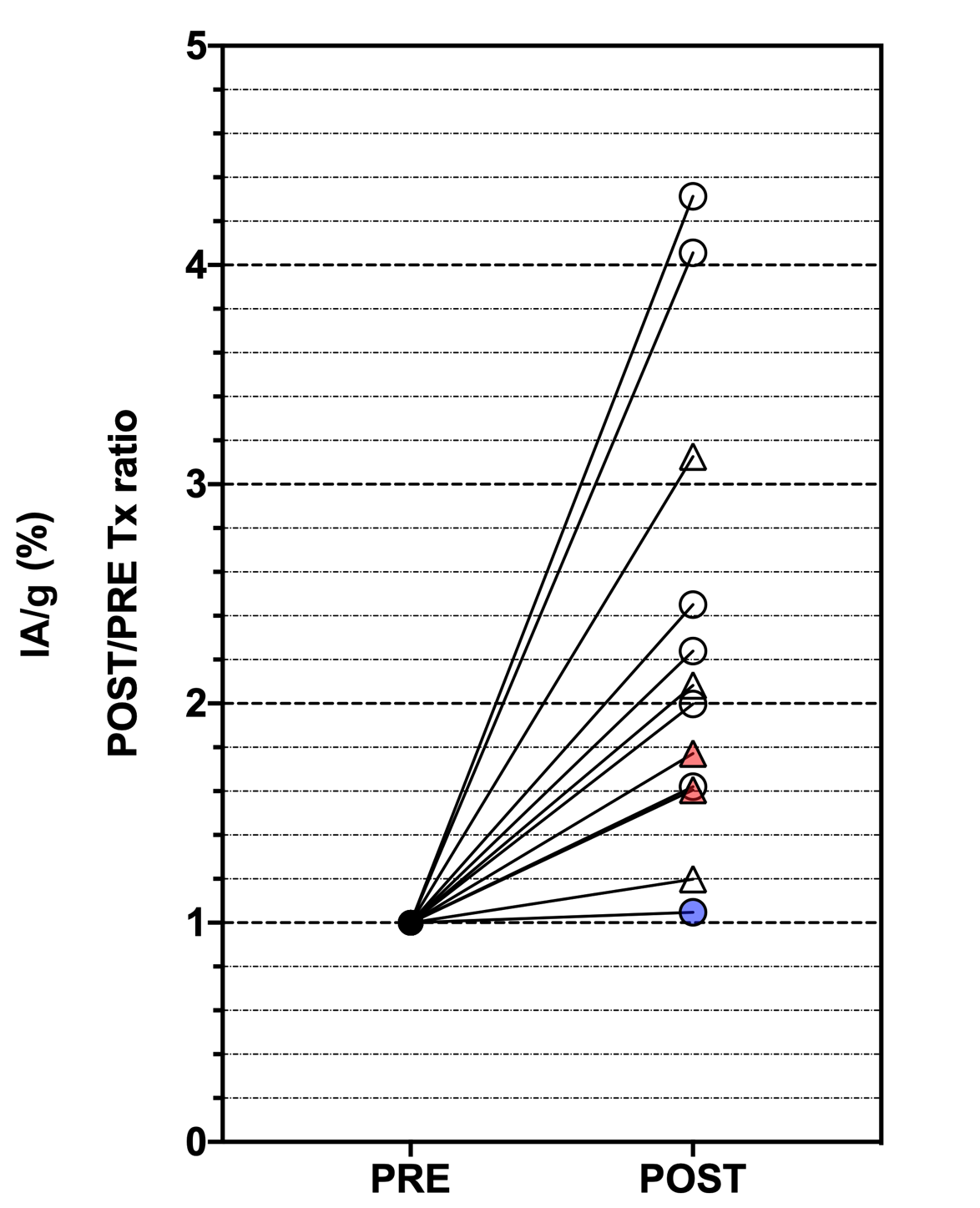


**Figure S11 –** ^18^F-C2Am tumor contrast in treated mice bearing MDA-MB-231 or Colo205 tumors, expressed as different metrics. *P* and *R* numbers in red, blue and black, correspond to Colo205, MDA-MB-231 and pooled data, respectively.

**
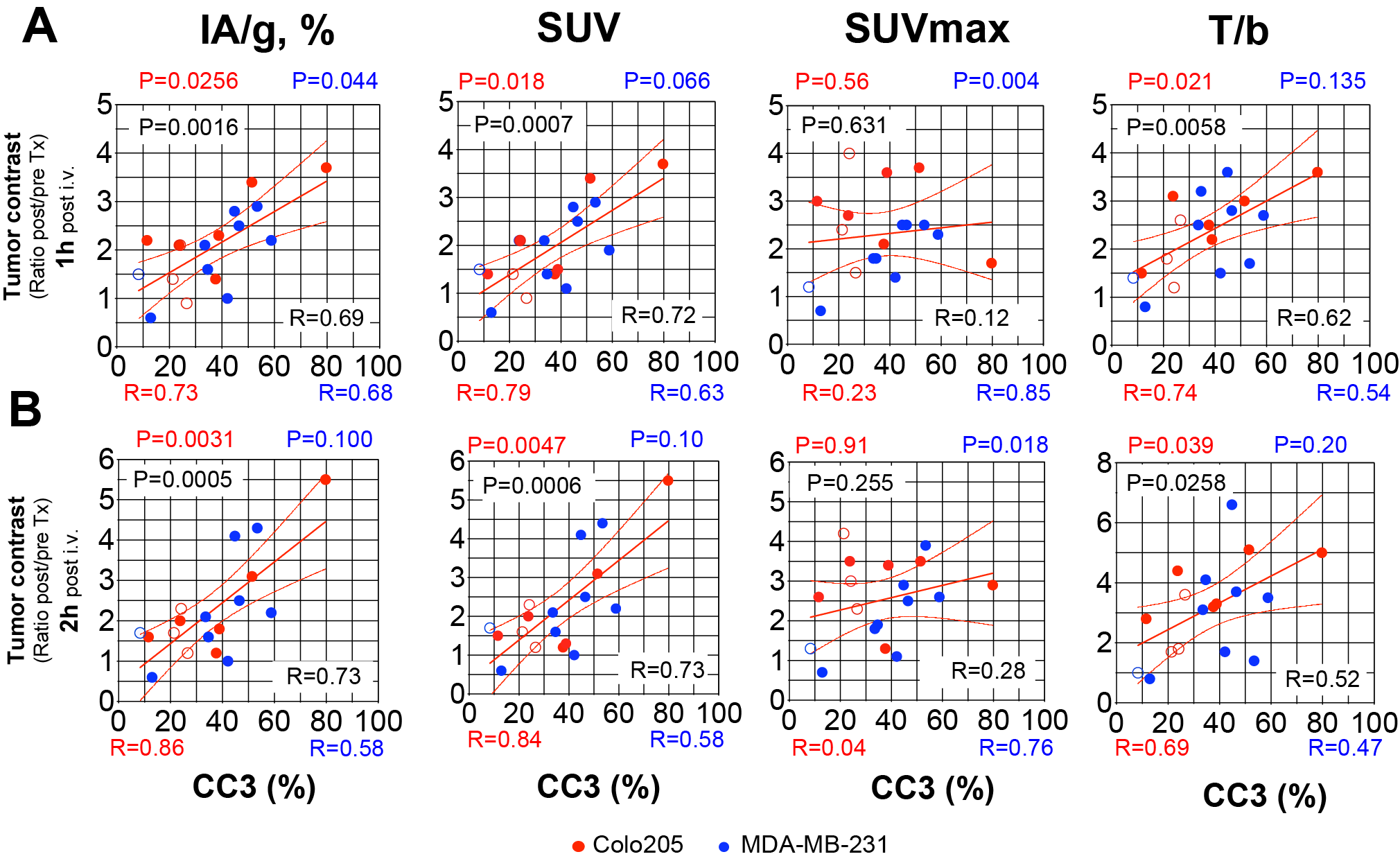
**

**Figure S12 –** Correlation analysis of the variation in ^18^F-C2Am tumor contrast before and after treatment, expressed as ΔSUV (A) or ΔSUVmax (B), as a function of the levels of tumor cell death (CC3, %) post treatment for both models. ΔSUV (%) =(SUVpost-SUVpre)/SUVpre x 100; ΔSUVmax (%) =(SUVmax_post-SUVmax_pre)/SUVmax_pre x 100, where pre and post denote treatment stage. Data shown for 1h (closed) and 2h (open circles) post injection of ^18^F-C2Am. Linear regression best-fit lines shown for 1h (thin) and 2h (thick line). Correlation parameters; ΔSUV, 1h: slope=2.74±0.36, R=0.694; 2h: slope=3.70±0.54, R=0.694; ΔSUVmax, 1h: slope=3.02±0.59, R=0.567; 2h: slope=3.68±0.62, R=0.339.

**Figure S13 –** Axial multi-slice PET/CT images showing distribution of ^18^F-C2Am in tumors post treatment for both models. Images were acquired before (PRE) and 24h post (POST) treatment with MEDI3039 (0.4 mg/kg) and 2h post injection of ^18^F-C2Am. Sequences correspond to multi-slice axial data of the mouse neck region, in a head to tail direction (left to right). Axial slice thickness was 0.4 mm. Arrows indicate start and end of tumor levels. ^18^F-C2Am signal shown as IA/g, %. Representative data shown for each tumor model.


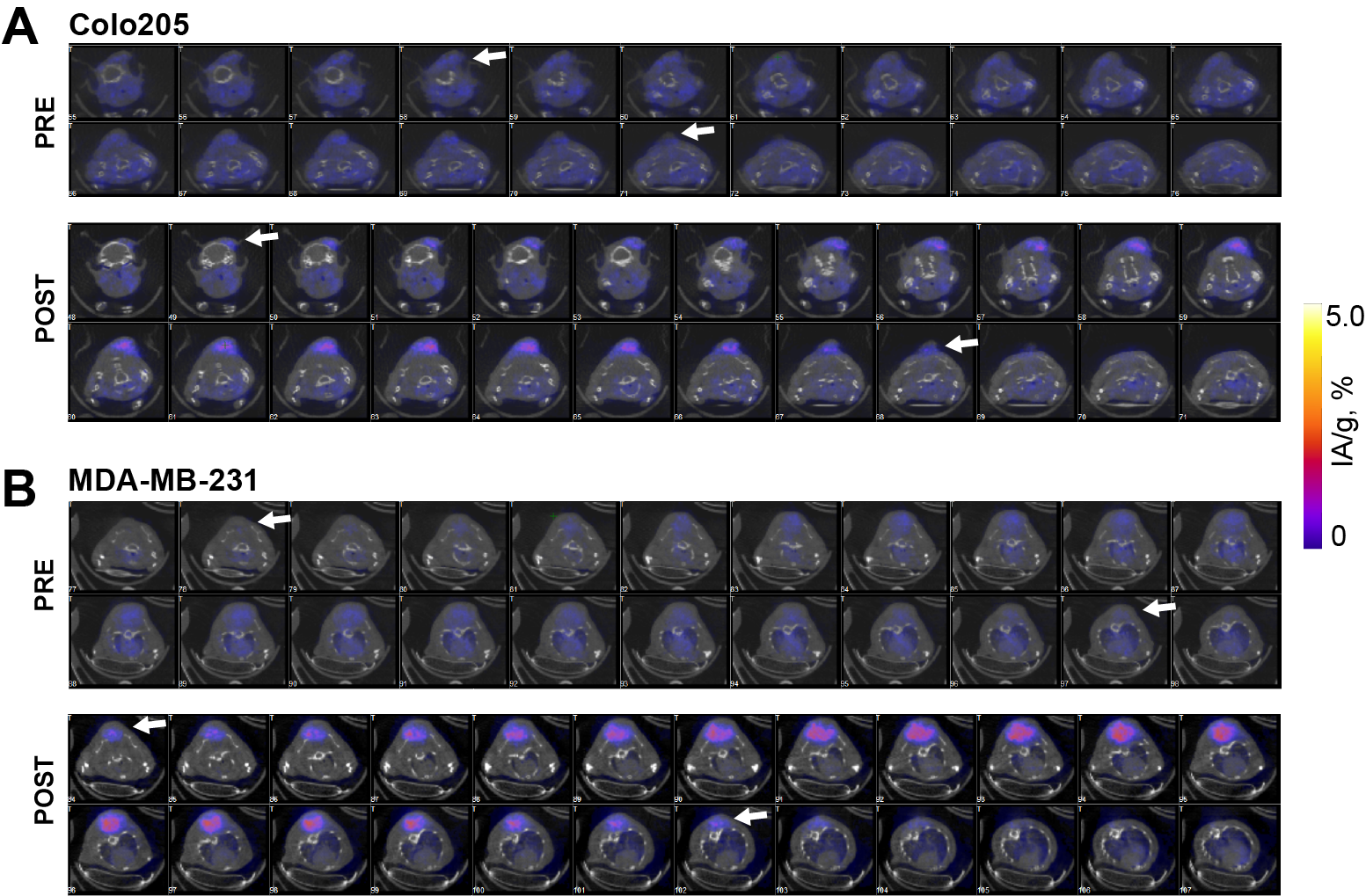


**Videos 1 and 2.** Dynamic PET/CT data of tumor cell death using ^18^F-C2Am, in MDA-MB-231 and Colo205 models, respectively. PET data shown as maximum intensity projection (M.I.P). CT was used to generate skeleton masks. Each video represents a concatenation of six video frames generated from dynamic data acquired from the same representative mouse, prior to and following treatment (MEDI3039, 0.4 mg/kg, i.v.). Time points shown: 0, 5, 15, 30, 60 and 120 minutes post injection of ^18^F-C2Am.

**References**

1. Alam IS, Neves AA, Witney TH, Boren J, Brindle KM. Comparison of the C2A domain of Synaptotagmin-I and Annexin-V as probes for detecting cell death. Bioconjug Chem. 2010;21:884-91.

2. Vanderhoek M, Perlman SB, Jeraj R. Impact of the definition of peak standardized uptake value on quantification of treatment response. J Nucl Med. 2012;53:4-11.

3. Xie B, Tomaszewski MR, Neves AA et al. Optoacoustic Detection of Early Therapy-Induced Tumor Cell Death Using a Targeted Imaging Agent. Clin Cancer Res. 2017;23:6893-903.

4. Hesketh RL, Wang J, Wright AJ et al. Magnetic Resonance Imaging Is More Sensitive Than PET for Detecting Treatment-Induced Cell Death-Dependent Changes in Glycolysis. Cancer Res. 2019;79:3557-69.

5. Coleman LE, Bork JF, Dunn H. Reaction of Primary Aliphatic Amines with Maleic Anhydride. J Org Chem. 1959;24:135-6.

6. Fujita Y, Murakami Y, Noda A, Miyoshi S. Design and Synthesis of an Easily Obtainable Maleimide Reagent N-[2-(4-[^18^F]fluoro-N-methylbenzenesulfonamido)ethyl]maleimide ([^18^F]FBSEM) to Radiolabel Thiols in Proteins. Bioconjug Chem. 2017;28:642-8.

7. Maina T, Konijnenberg MW, KolencPeitl P, Garnuszek P, Nock BA, Kaloudi A, Kroselj M, Zaletel K, Maecke H, Mansi R, Erba P, von Guggenberg E, Hubalewska-Dydejczyk A, Mikolajczak R, Decristoforo C. Preclinical pharmacokinetics, biodistribution, radiation, dosimetry and toxicity studies required for regulatory approval of a phase I clinical trial with (111)In-CP04 in medullary thyroid carcinoma patients. Eur J Pharm Sci. 2016;91:236-42.

8. Andersson M, Johansson L, Eckerman K, Mattsson S. IDAC-Dose 2.1, an internal dosimetry program for diagnostic nuclear medicine based on the ICRP adult reference voxel phantoms. EJNMMI Res. 2017;7:88.
